# Succinylated nori protein–alginate complex particles prepared using temperature-controlled ultrasonication for the formation of high internal phase Pickering emulsion gels as plant-based fat analogues^☆^

**DOI:** 10.1016/j.ultsonch.2026.108029

**Published:** 2026-08-26

**Authors:** Xinxin Si, Zhuozhen Li, Yixi Chen, Bingheng Li, Li Zhang, Jiefu Hai, Yichen Zhang, Boyao Su, Xiao Feng, Tian Zhong, Ying Xiao, Xi Yu

**Affiliations:** a Faculty of Chinese Medicine, Macau University of Science and Technology, Taipa, Macao 999078, China; b Faculty of Medicine, Macau University of Science and Technology, Taipa, Macau; c Department of Food Science, Zhaoqing University, Zhaoqing, China; d Faculty of Science, The University of Hong Kong, Baiyun District, Guangzhou, China; e College of Food Science and Engineering/Collaborative Innovation Center for Modern Grain Circulation and Safety/Key Laboratory of Grains and Oils Quality Control and Processing, Nanjing University of Finance and Economics, Nanjing, China; f Department of Environmental Health, Harvard T.H. Chan School of Public Health, Boston, USA; g Zhuhai MUST Science and Technology Research Institute, Zhuhai, China

**Keywords:** Ultrasonication, Succinylation, Complex particles, High-internal-phasePickering emulsion gels, Plant-Based fat analogues

## Abstract

In this study, succinylated nori protein (SNP), sodium alginate (SA), and flaxseed oil were used to develop novel high-internal-phase Pickering emulsion (HIPPE) gels with potential application as plant-based fat analogues. Stable composite particles were successfully fabricated through ultrasonication combined with temperature control. The ultrasonic treatment condition was identified as 450 W for 30 min, under which the Pickering particles exhibited the smallest particle size and superior emulsifying ability. The optimal formulation parameters were identified as an SNP-to-SA ratio of 1:5, an SNP-SA complex particle concentration of 3%, and an oil-phase fraction of 75%, which resulted in optimal oil retention and emulsion stability. Confocal laser scanning microscopy and cryo-scanning electron microscopy revealed that the SNP-based emulsion produced smaller droplets (23.08 ± 0.09 μm) and a more compact core–shell interfacial structure than the emulsion prepared with native nori protein. These structural improvements were associated with enhanced adsorption kinetics and wettability, with a contact angle approaching 90°, indicating optimal amphiphilicity. Rheological analysis further demonstrated that the emulsion exhibited more solid-like viscoelastic behavior, with a greater difference between G′ and G″ than that prepared with native nori protein. Moreover, microscopic observations showed that the dense packing of lipid droplets within the gel network resembled the cellular architecture of animal fat tissue. The thermal behavior of the HIPPE gels in the range of 200–500 °C was similar to the two downward endothermic peaks observed for pork fat during cooking. Sensory and texture analyses of meat patties revealed that 25% and 50% fat substitution levels could closely mimic full-fat meat patties. Overall, the proposed material provides an environmentally friendly and healthy strategy for developing fat analogues from marine-derived protein resources and flaxseed oil, while mimicking animal fat tissue in terms of texture, microstructure, thermal behavior, and sensory properties.

## Highlights

• Ultrasonic cavitation breaks down aggregates into smaller, uniformly dispersed particles with superior emulsifying ability.
• Succinylation modification increase the hydrophilicity and change the spatial structure of NP.
• SNP-SA densely packed particles with the amphiphilicity form the highly stable and dispersed HIPPE.
• The gel has superior texture characters and higher elasticity, analogous to animal fat tissue.
• The gel microstructure with lipid droplets have promising ability to mimic animal fat cell.

## Graphical abstract

**Figure:**
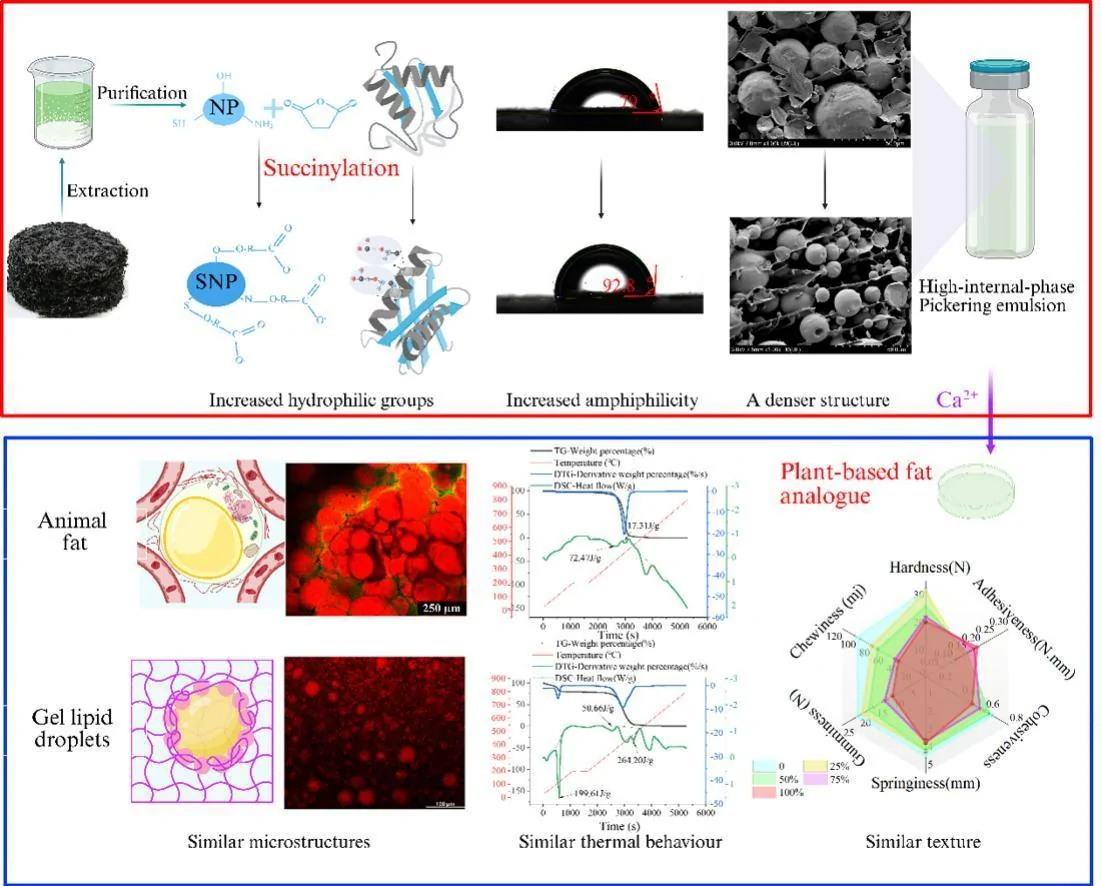

## Introduction

Animal fat is widely used in meat products because it provides characteristic flavor, mouthfeel, high energy density, and satiety. However, its high content of saturated fatty acids has been associated with increased risks of cardiovascular disease, atherosclerosis, type 2 diabetes, and obesity [1], [2]. In addition, growing concerns about the environmental impact of livestock production have further driven the search for healthier and more sustainable fat analogues. Current strategies mainly include the incorporation of protein- and polysaccharide-based fat analogues into meat systems to improve fat distribution and reduce saturated fat content, as well as the production of synthetic or structured fats through chemical modification methods such as hydrogenation and interesterification [3]. However, these approaches may involve several limitations, including gastrointestinal discomfort, impaired vitamin absorption, and the possible presence of trans or excessive saturated fatty acids. Therefore, the structuring of plant oils into solid-like plant-based fat analogues has emerged as an attractive alternative. Among these approaches, emulsion gels have shown particular promise because they can mimic the texture of animal fat while providing a higher proportion of unsaturated fatty acids and improved health benefits [4], [5]. As a result, emulsion gels are increasingly being explored not only as fat analogues but also as a versatile platform for the development of plant-based meat analogues.

Pickering emulsions (PEs), which are stabilized by solid particles rather than conventional surfactants, were first reported in the early 20 century [6]. With the continuous development of this field, high-internal-phase Pickering emulsion (HIPPE) gels, defined as emulsions containing an internal phase volume fraction exceeding 74%, have emerged as a major research focus in recent years [7]. Their ability to transform liquid vegetable oils into solid or semi-solid systems without altering the fatty acid composition offers a promising strategy for mimicking animal fat. Numerous studies have shown that HIPPE gels possess solid-like characteristics, high oil loading, and excellent physical stability. In addition, they are considered healthier clean-label alternatives because of their lower contents of saturated fatty acids and cholesterol. However, the texture and functionality of HIPPE gels are strongly dependent on the properties of their emulsion precursors, which are, in turn, governed by the characteristics of the Pickering particles used for stabilization.

Pickering particles (PPs) irreversibly adsorb at the oil–water interface, forming a dense interfacial barrier that resists droplet coalescence and Ostwald ripening, thereby conferring stability, tunable texture, and shear-thinning behavior to emulsions [8]. The focus of Pickering particle research has gradually shifted from synthetic materials to edible biomacromolecules. Among these, plant-derived colloidal particles based on proteins and polysaccharides are particularly attractive because they are non-toxic, sustainable, and biocompatible, making them promising stabilizers for high-internal-phase Pickering emulsions (HIPPE) [9], [10]. For example, Wen et al. reported that HIPPE gels prepared using soy protein nanoparticles at different concentrations exhibited varying degrees of self-supporting properties. Peng et al. further demonstrated that HIPPE stabilized by binary protein-polysaccharide conjugates exhibited a three-phase contact angle closer to 90°, which is considered favorable for emulsion stability. Increasing evidence suggests that binary composite particles often offer more pronounced advantages than single-component biomacromolecules [11], [12]. Recent studies have confirmed that protein or polysaccharide modification can also significantly improve emulsion stability, underscoring the view that native biomacromolecular structures are not the endpoint, but rather the starting point for functional innovation [13], [14]. Nevertheless, several challenges remain unresolved. First, large-scale production of terrestrial plant proteins competes with food crops for land and water, raising sustainability concerns. Second, the mechanisms underlying the enhanced stability provided by multicomponent particle systems remain insufficiently validated experimentally. Third, many plant proteins exhibit strong intrinsic hydrophobicity and rigid secondary structures, which hinder the attainment of an optimal contact angle close to 90°. Finally, the long-term storage stability of plant-based fat analogues and 3D-printing inks remains inferior to that of conventional edible fats and oils, limiting their practical application.

**Fig. 1:**
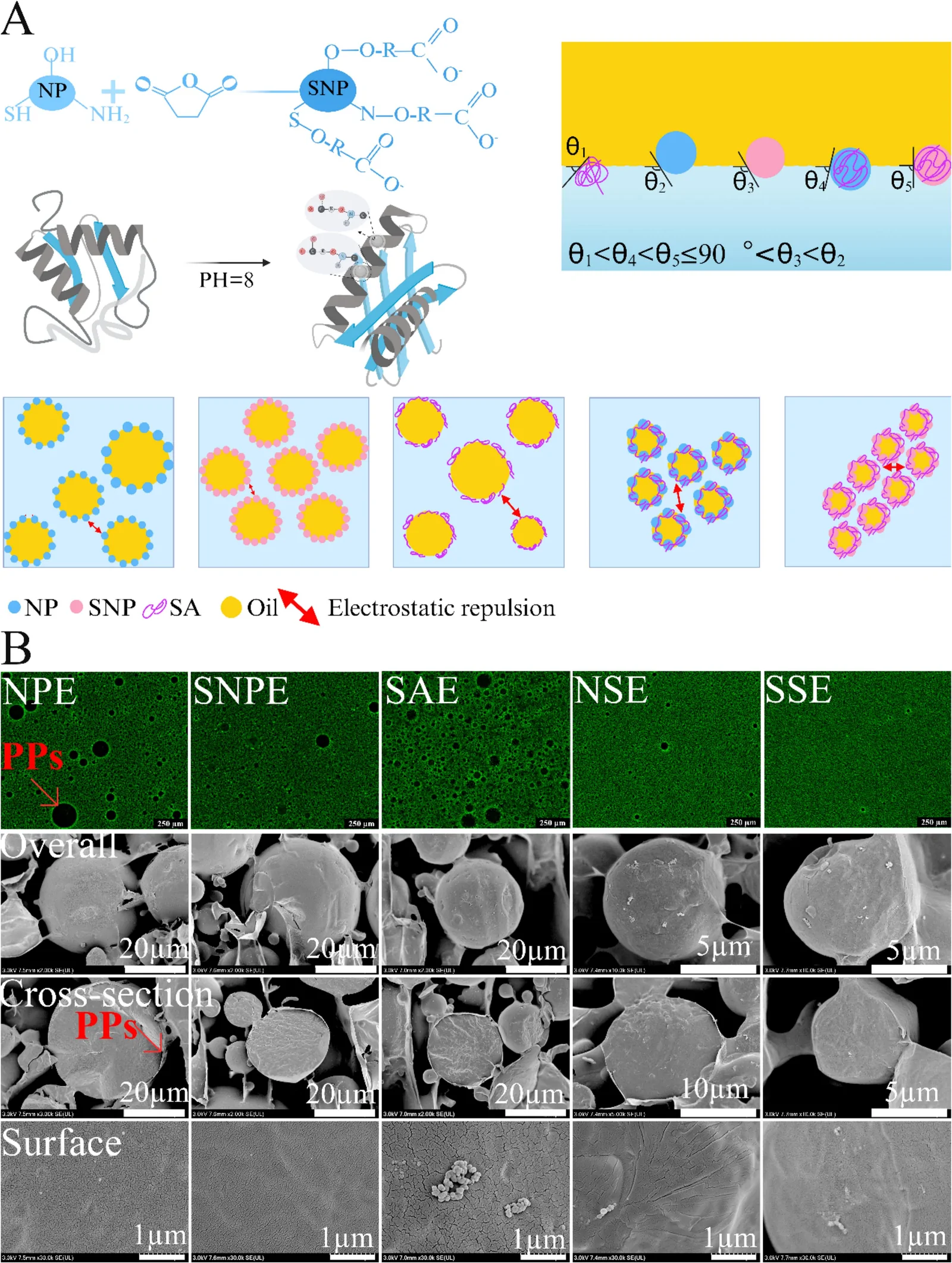
Mechanisms for the formation and stability of Pickering emulsion with individual and binary composite particles. (A) Mechanism diagram for the formation of Pickering emulsion. (B) Surface and cross-sectional views of the formed Pickering emulsion.(NP: protein particles, SNP: succinylated protein particles, SA: sodium alginate particles, NS: sodium alginate and protein composite particles, SS: sodium alginate and succinylated protein composite particles. NPE: nori protein particle-stabilized emulsion. SNPE: succinylated nori protein particle-stabilized emulsion. SAE: sodium alginate particle-stabilized emulsion. NSE: sodium alginate and protein composite particle-stabilized emulsion. SSE: sodium alginate and succinylated protein composite particle-stabilized emulsion.PPs: Pickering particles).

Nori (*Porphyra yezoensis*), a type of red algae, is rich in protein, with R-phycoerythrin being particularly abundant and demonstrating significant antioxidant, immunomodulatory, and anticancer effects [15]. With an annual production exceeding one million tons, nori serves as a sustainable natural resource and shows great potential as an alternative to animal-derived and terrestrial plant-based proteins [16], [17]. To date, research on marine-derived plant-based materials has focused mainly on marine polysaccharides. For instance, Zhang et al. reviewed the potential of marine polysaccharides as Pickering particles, while Meng et al. successfully fabricated Pickering particles from chitosan and seaweed polyphenols [18], [19]. Compared with terrestrial plant proteins, the application of seaweed proteins in emulsion systems remains largely unexplored. A survey of the available literature indicates that only a limited number of studies have examined their roles in emulsion stabilization, and even fewer have addressed their interfacial properties, highlighting the novelty of the present work. Studies on the modification of marine proteins are also still extremely rare. Succinylated nori protein (SNP) and sodium alginate (SA) represent a novel marine-derived composite system with considerable potential as sustainable natural colloidal stabilizers for HIPPE [20]. Succinylation was carried out to overcome the limitations of native nori protein as a Pickering stabilizer. Although native protein can participate in particle formation, its surface charge, conformation, and interfacial wettability are not optimal for stabilizing HIPPE (Fig. 1A). By introducing additional carboxyl groups, succinylation increases the negative charge density and modifies the protein structure, which enhances dispersibility, electrostatic repulsion, and interfacial adsorption behavior [21]. Molecular docking results reported previously, showing increased binding sites for cationic polymers, further support this interpretation [22], [23]. Therefore, this study aimed to combine protein modification and binary complexation to construct highly stable and structurally refined HIPPE gels. The electrostatic interactions and successful complexation between SNP and SA were systematically confirmed by FTIR, XRD and particle size. As shown in Fig. 1B, compared with single particles, the composite particles formed a denser core–shell structure at the oil droplet interface and produced smaller droplets overall. Similarlly, the combination of hydrophobic succinylated protein domains and hydrophilic SA chains produced amphiphilic particles with a three-phase contact angle close to 90° (Fig. 1A) [24]. In addition, the resulting HIPPE exhibited improved microstructure, rheological properties, Interfacial adsorption behavior, and pH/temperature storage stability, indicating its suitability as a precursor for oleogel preparation. Herein, we report a timely and potentially valuable material, HIPE gels, stabilized by modified marine-derived composite particles, which exhibit greater toughness and flexibility than those based on terrestrial proteins, along with a high oil content for mimicking animal fat. The HIPPE-based gel exhibited promising capability in textural properties, microstructurethermal behavior and sensory properties to mimicing those of animal fat. Overall, this work establishes a new HIPPE gel with SNP-SA composite particles as a novel marine-derived and sustainable strategy for mimicking animal fat and provides a new avenue for the development of plant-based meat products in fat apply.

## 2 Materials and methods

### 2.1 Materials

Nori and Flaxseed oil were purchased from Tmall Supermarket. Pork fat tissue was purchased from Xinmiao Supermarket in Macau Sodium alginate from Yuanye Biotechnology Co., Ltd. (Shanghai, China). Nile red and fluorescein isothiocyanate (FITC) were supplied by Shanghai Macklin Biochemical Technology Co., Ltd. (Shanghai, China). Succinic anhydride and sodium dodecyl sulfate (SDS) were purchased from Solarbio Corporation in Beijing, China. The dialysis membrane (MD 44, MW 3500) was purchased from Beijing Sun Biotechnology Co., Ltd. All the orher chemical reagents were analytical grade reagents from nanjing jiancheng bioengineering institute.

### 2.2 Ultrasonication of protein–polysaccharide composite particles

Nori protein (NP) was extracted by an ultrasound-assisted alkali extraction–acid precipitation method (Ultrasound bath, LICHEN, LC-UC-220, Shanghai, China) and subsequently modified by succinylation. Detailed experimental procedures are described in the Supplementary Materials and Fig. S1. Freeze-dried NP, succinylated nori protein (SNP), and sodium alginate (SA) were each dispersed in deionized water at 30 mg/mL. The pH of the NP and SNP dispersions was then adjusted to 2.0, and each protein dispersion was mixed with the ultrasonicated SA solution (pH 7.0) at a protein-to-polysaccharide mass ratio of 1.5:1 [25]. The obtained mixtures were designated as NS (NP-SA complex) and SS (SNP-SA complex), respectively. After electrostatic complexation for 10 min, the pH of the mixtures was readjusted to 7.0, followed by ultrasonication at 0–550 W for 30 min at 50 °C. Similarly, the composite particles were subjected to ultrasonic treatment at 450 W for 10, 20, 30, 40 and 50 min, respectively. The particle size and zeta potential using a Zetasizer Nano ZS (Malvern, Zetasizer Lab, UK), were subsequently determined [26], [27].

### 2.3 Fourier transform infrared spectroscopy (FTIR) and X-ray diffraction (XRD) of Pickering particles

The freeze-dried sample was scanned 64 times over the wavenumber range of 400–4000 cm^−1^ at a resolution of 4 cm^−1^, and the transmission spectra were baseline-corrected and smoothed. The FTIR (Shimadzu, Iraffnity-1S, Japan) spectra were modeled and fitted using PeakFit v4.12 software. For the amide I region (1600–1700 cm^−1^), baseline smoothing, deconvolution, and second-derivative fitting were performed to resolve the spectra into multiple component peaks. Fourier deconvolution was applied to the 1600–1700 cm^−1^ spectral region to determine the protein secondary structure. The relative proportions of these secondary structures were calculated based on the PeakFit fitting results [28].

The crystal structure was analyzed by powder X-ray diffraction (XRD, D8 Advance, Germany). Data were collected over a 2θ range of 5-80° with Cu Kα1 radiation (λ = 1.54056 Å) operating at 40 kV and 40 mA. Analysis was performed using MDI Jade 9 software with reference to the PDF-4 (2009) database.

### 2.4 Preparation of Pickering emulsions and gels

Pickering composite particles were prepared by varying the particle concentration, oil phase fraction, and protein-polysaccharide ratio. The resulting mixtures were homogenized at 10,000 rpm for 2 min (BIAOBEN, FJ200, Shanghai, China), transferred into glass vials (22 mm in width × 50 mm in height), allowed to stand, and photographed at designated time intervals.

HIPPE containing particle concentrations of 3%, 4%, 5%, and 6% were placed into molds and immersed in 0.1 mol/L CaCl_2_ solution for 48 h to induce gelation. For fluorescence imaging, 1.5 g of each HIPPE was transferred into a 2 mL centrifuge tube and stained with FITC and Nile Red solutions. The stained samples were then immersed in 0.1 mol/L CaCl_2_ solution until solid gels formed. Subsequently, 1 cm × 1 cm gel sections were cut and examined under a confocal laser scanning microscope for microstructural observation.

### 2.5 Microstructure observation

To elucidate the microstructure of the Pickering emulsions with different types of composite particles, confocal laser scanning microscopy (CLSM, Leica, VL2000DX-SVF17SP, Japan) was employed for observation at 25 °C using a 20 × and 60 × objective lens. For phase differentiation, 0.1% FITC (dissolved in isopropanol) was used to stain nanoparticles in the continuous phase (green), and Nile red was used to stain the oil phase (red). The excitation/emission wavelengths were set at 550/600–670 nm for Nile red and 488/500–561 nm for FITC. The emulsion sample was mixed with the dye solution at a volume ratio of 50:1, and a drop of the mixture was placed on a glass slide, gently covered with a coverslip, and then imaged [29].

The Cryo-field emission scanning electron microscopy (Cryo-SEM, FEI Quanta 450, Thermo Fisher Scientific, USA) process begins with coating the sample stage with a cryo-compatible conductive adhesive and attaching the sample using tweezers. The sample, now securely affixed, undergoes rapid freezing in liquid nitrogen slush for 30 s. Subsequently, it is transferred under vacuum using a low-temperature cryo-transfer system to a sample preparation chamber. Inside the chamber, the sample undergoes sublimation at −90 °C for 10 min to remove any surface contaminants. Then, gold is sputtered onto the sample at 10 mA for 60 s to enhance its conductivity. It was sent into the sample chamber of the scanning electron microscope. The cold stage temperature is −140℃, and the accelerating voltage is 5 kv. Observe at magnifications of 1 K×, 2 K×, 10 K × and 30 K × .

### 2.6 Wettability of the particles and measurement of interfacial tensions

The water contact angles of the NS and SS were measured using a contact angle analyzer (OCA-15EC, Dataphysics Co., Germany). Freeze-dried samples were first compressed into thin films using a hydraulic press. Each film was then immersed in linseed oil, and a 3 µL droplet of deionized water was deposited onto its surface using a high-precision syringe [30].

The interfacial tensions of water-in-oil droplets were measured by pendent drop experiments at room temperature (Drop Meter A-200, Ningbo NB Scientific Instruments Co., Ltd.). The adhesion and coalescence behavior between droplets was evaluated using a force tensiometer (Dataphysics Instruments, DCAT 25, Germany). Aqueous droplets were generated and positioned vertically, with one droplet suspended from the upper probe and the other placed on the lower stage. The upper droplet was carefully lowered to approach, contact, and compress the lower droplet, after which it was retracted to separate them. The entire process of droplet contact and separation was recorded using a high-speed industrial camera. To ensure image clarity, uniform illumination and a clean background were maintained throughout the measurements [31], [32]. The aqueous phase consisted of 30 mg/mL of NS and SS.

### 2.7 Rheological measurements of high internal phase Pickering emulsions (HIPPE)

Based on the emulsion stability analysis presented in the Supplementary Materials, a high internal phase Pickering emulsion (HIPPE) with an oil content of 75% (w/w) was prepared using the optimal formulation, consisting of 3% (w/w) protein-polysaccharide particles at a mass ratio of 1:5. Rheological measurements were performed using a dynamic rheometer (DHR 1, Waters, USA) equipped with a P35/Ti parallel plate geometry (1 mm gap). For each measurement, 1 mL of emulsion was loaded onto the lower plate, and the sample was allowed to equilibrate at the test temperature. Three distinct rheological protocols were applied, all within the linear viscoelastic region (LVR): **Strain sweep**: Oscillatory amplitude sweeps were performed at a fixed angular frequency of 1.592 Hz (10 rad/s), with strain amplitudes ranging from 0.01% to 100%. **Steady shear**: Flow sweeps were conducted over a shear rate range of 0.1 to 100 s^−1^. **Frequency sweep**: Oscillatory frequency sweeps were carried out over an angular frequency range of 0.1 to 100 rad/s, using a constant strain of 50% (within the LVR).

### 2.8 Temperature and pH responsiveness of HIPPE

The pH and temperature stability of the HIPPE was assessed by adjusting the emulsions to various pH values (using 0.1 M NaOH or HCl) and incubating each sample at different temperatures for 20 min, followed by photography using an optical microscope (AOSVI, CM30-Y90, Shenzhen, China). The droplet size distribution and ζ-potential of the Pickering emulsions was determined using a laser particle size analyzer (Malvern Mastersizer 2000, Malvern Instruments, UK), and the volume-weighted mean diameter (D [4,3]) was reported. Measurements were based on Fraunhofer diffraction theory. Prior to analysis, each sample was diluted 100-fold with ultrapure water. The refractive indices were set to 1.479 for the oil phase (perilla oil) and 1.333 for the dispersant (water). The droplet size measurement range was configured as 0.5–100 μm [33].

### 2.9 Determination of HIPPE gels strength

Gel strength of the HIPPE gels were performed using a texture analyzer (TA-CT3, Brookfield Engineering Laboratories, Inc., Middleboro, MA, USA). For gel strength measurement, a 12 mm cylindrical probe was penetrated into the sample to a depth of 8 mm.

### 2.10 Freeze-thaw stability and thermogravimetry–differential scanning calorimetry (TG-DSC)

Freeze-thaw stability of the HIPPE gels was determined using a modified version of the method described by [34]. Samples in Petri dishes were subjected to five cycles of freezing at −20 °C for 20 h and thawing at 25 °C for 30 min. Syneresis was quantified by measuring the weight loss after each cycle, and Syneresis (%) was calculated using the following equation (1):

(1) $$Syneresis\;\left(\%\right)=\frac{m_{b}\;-{\;m}_{a}}{m_{b}}\times 100$$

where m_b_ and m_a_ are the weights of the emulsion gel before and after freeze–thaw treatment, respectively.

The thermal stability of the emulsion gels was evaluated using simultaneous thermogravimetry–differential scanning calorimetry (TG-DSC) (STA 409 PC, Netzsch, Germany). Approximately 5–10 mg of each sample was heated from 30 °C to 800 °C at a rate of 10 °C/min under a constant nitrogen flow of 100 mL/min. A holding time of 10 min was applied at 200 °C. Thermal analysis curves were recorded for further interpretation.

### 2.11 Determination of performance characterization of meat patties

#### 2.11.1 Preparation of meat patties with different content of fat analogues

The preparation of pork patties was conducted according to the method of Bai et al. [35], with slight modifications [35]. Cleaned Lean pork meat and pork back fat were minced at a lean-to-fat ratio of 8:2 using a meat grinder (Grindomix GM 200, RETSCH, Germany) equipped with a 6 mm grinding plate for 2 min. Five patties formulations were prepared, in which pork backfat was replaced at 0%, 25%, 50%, 75%, and 100% of the total fat content. Salt, sugar, sodium tripolyphosphate, red yeast rice, and corn starch were then added, and the mixture was chopped for a further 2 min, with a 1 min pause during processing. Subsequently, pork back fat, half of the ice water, the fat analogues, and sodium erythorbate were incorporated and chopped for another 2 min. The remaining ice water was then added, followed by an additional 2 min of chopping. The resulting batter was shaped into patties with a diameter of 7 cm and a thickness of 1.8 cm. The raw patties were pan-fried for 20 min until the internal temperature reached 72 °C. After cooking, the low-fat patties samples were cooled and stored at 4 °C prior to subsequent analyses. Detailed formulation information is provided in Table S2.

#### 2.11.2 Appearance and color determination

The appearance of the patties was evaluated by capturing digital images using a smartphone. The colour of the samples before and after cooking was measured using a colorimeter (NR10QC, 3nh, China) [36].

#### 2.11.3 Cooking loss

The patties were weighed before cooking. After pan-frying, the samples were cooled at room temperature for 2 h, and cooking loss was then determined according to the method described by Zhang et al [37].

(2) $$Weight\;loss=\frac{m_{a}-m_{b}}{m_{a}}$$

### 2.11 4 Determination of TPA for meat patties

TPA of the HIPPE gels were performed using a texture analyzer (TA-CT3, Brookfield Engineering Laboratories, Inc., Middleboro, MA, USA). For TPA, the following parameters were applied: trigger force of 0.75 N, deformation of 30%, and test speed of 20 mm/min.

#### 2.11.5 Sensory evaluation

The sensory panel consisted of 15 trained assessors, who were recruited and trained according to the methods described by Nie et al. [38], [38]The prepared patties were accurately cut into cubes (1 × 1 × 1 cm^3^), placed on white plates, and presented to consumers for preference testing using three-digit random codes. Participants were instructed to rinse their mouths with room-temperature water between samples and were not allowed to communicate with each other during the evaluation. The patties prepared with 100% pork backfat was used as the reference sample and assigned a score of 5. Based on comparisons between the reference sample and the other experimental groups, the assessors evaluated the patties for acceptability, umami, meaty flavour, off-odour, flavour, hardness, chewiness, springiness, juiciness, mouthfeel, redness, yellowness, porosity, cut-surface smoothness, and appearance using a scale ranging from 0.0 to 10.0. The same sensory evaluation was repeated on different working days by the same group of assessors [38].

### 2.12 Statistical analysis

Data were analyzed using OriginPro (Version 2021, OriginLab Corporation). All measurements were performed in at least triplicate, and results are reported as mean ± SD. Statistical significance among groups was evaluated by one-way ANOVA followed by the Holm-Šídák test, with *P* < 0.05 considered significant.

## 3 Results and discussions

### 3.1 Characterization of native protein and succinylated protein

Nori protein (NP) was successfully extracted using an alkali extraction–acid precipitation method, and its protein content was quantified by the Bradford assay using BSA as the standard (Fig. S1A). The crude protein extraction yield was 19.0 ± 0.79%, with a protein purity of 73.68 ± 0.97%, indicating that the extraction method was effective for obtaining relatively protein-rich fractions. After succinylation, the degree of modification increased progressively and reached a maximum of 74.37 ± 1.2% at an acylation reagent concentration of 10% (Fig. S1B), consistent with the findings of Dnyaneshwar Patil et al. [39]. Succinylation exposed more hydrophilic groups on the protein surface; nevertheless, the resulting three-phase contact angle was still lower than 90°. After complexation with SA, the composite particles displayed a contact angle closer to 90°, indicating improved amphiphilic wettability (Fig. 1A). This result was in good agreement with the subsequent contact-angle measurements. In addition, Fig. 1B aslo shows that emulsions stabilized by composite particles had smaller droplet sizes than those stabilized by single particles, while emulsions stabilized by succinylated protein particles exhibited smaller droplets than those stabilized by native protein particles.

To elucidate the role of amino acid composition in Pickering emulsion formation, the amino acid profiles and contents of the proteins were determined. The amino acid compositions of NP and succinylated NP (SNP) are shown in Fig. S1C. The predominant amino acids were aspartic acid (Asp), glutamic acid (Glu), arginine (Arg), alanine (Ala), and leucine (Leu), representing acidic, basic, and hydrophobic amino acid groups. NP contained 2.49 ± 0.26% lysine (Lys), which is of particular interest because lysine residues are the primary sites for succinylation. Hydrophobic amino acids accounted for 46.07 ± 0.26% and 48.11 ± 0.37% of the total amino acid content in NP and SNP, respectively, indicating the hydrophobic character of both proteins. This further confirms the above conclusion that both nori protein and the modified nori protein exhibit hydrophobic characteristics. This intrinsic hydrophobicity was further supported by the absence of oil separation in the upper layer of the emulsions during long-term storage. Notably, the overall amino acid composition remained essentially unchanged after succinylation, suggesting that the improved emulsifying properties were mainly attributable to alterations in protein conformation and surface charge rather than changes in amino acid composition.

### 3.2 Characterization of the Pickering complex particles

#### 3.2.1 Particle size and zeta potential of complex particles prepared under different ultrasonic conditions

**Fig. 2:**
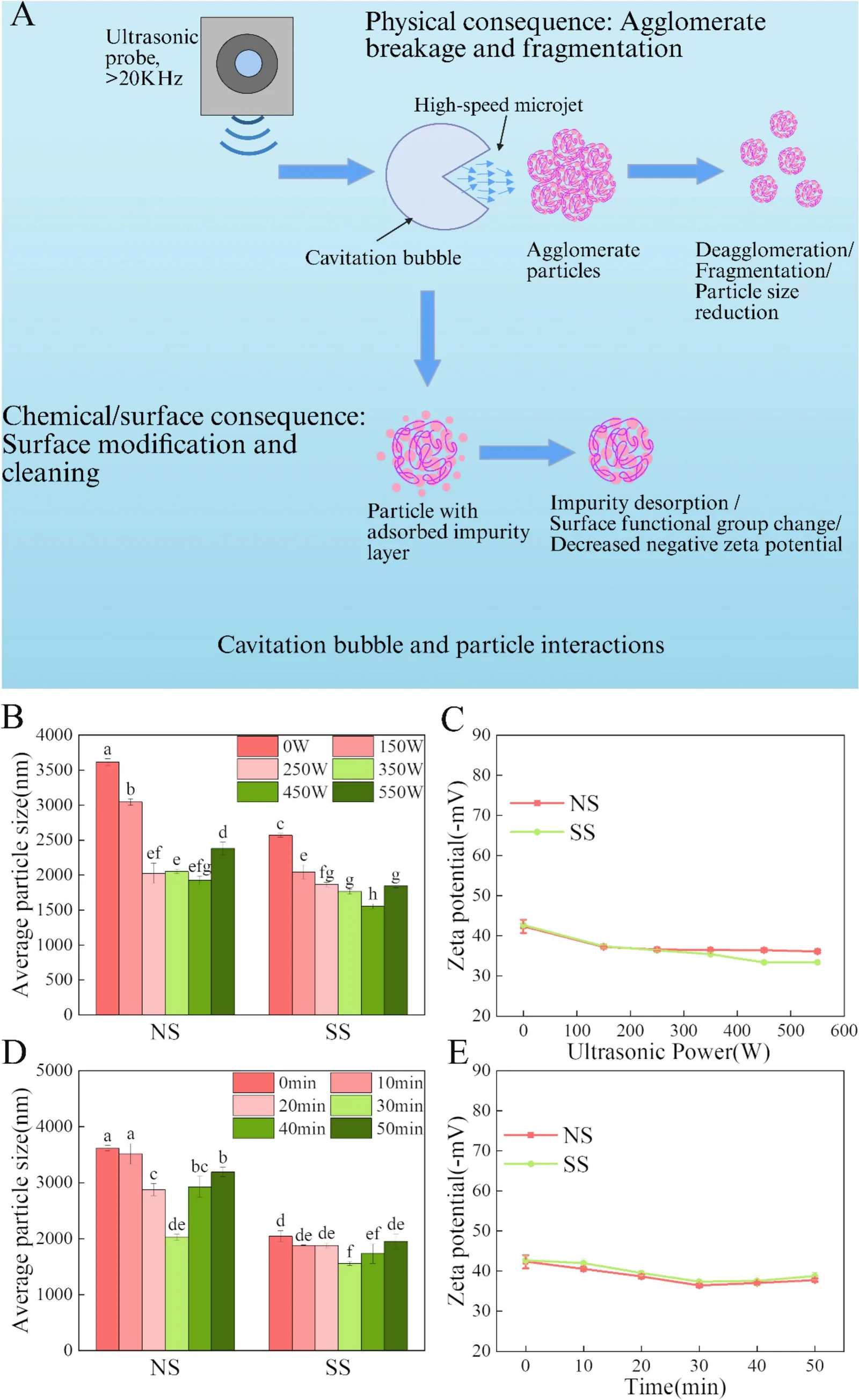
Effects of ultrasonication on Pickering particles. (A) particle size of composite particles under different ultrasonic power levels; (B) zeta potential of composite particles under different ultrasonic power levels; (C) particle size of composite particles under different ultrasonication times; (D) zeta potential of composite particles under different ultrasonication times. (NS: sodium alginate and protein composite particles, SS: sodium alginate and succinylated protein composite particles.).

The particle size and surface charge of Pickering particles play crucial roles in determining their behavior at the oil–water interface and, consequently, the stability of Pickering emulsions. Smaller particles provide a larger specific surface area and higher diffusion rates, enabling more efficient interfacial adsorption, improved surface coverage, and enhanced steric stabilization. In parallel, a sufficiently high zeta potential contributes to emulsion stability by generating electrostatic repulsion between droplets. As shown in Fig. 2B and D, the prepared composite particles exhibited average size of 3616.89 ± 48.22 nm for NS and 2569.76 ± 27.19 nm for SS. With increasing ultrasonic power, the particle size decreased progressively, reaching its minimum at an ultrasonic power of 450 W and a treatment time of 30 min. This reduction in particle size can be attributed to the cavitation effect induced by ultrasonication, which generates cavitation bubbles and intense shear forces [40]. As illustrated in Fig. 2A, the interaction between ultrasonic cavitation bubbles and particles can physically disrupt unfolded biomacromolecular aggregates, thereby breaking down the aggregated structures into smaller and more uniformly dispersed particles.

In addition, the absolute value of the zeta potential decreased significantly after ultrasonication (Fig. 2C and E). At pH 7.0, the zeta potential of ultrasonically treated particles ranged from − 37.25 ± 0.13 to − 36.11 ± 0.29 mV for NS and from − 33.43 ± 0.25 to − 37.41 ± 0.25 mV for SS, whereas the corresponding untreated particles exhibited higher absolute zeta potential values of − 42.37 ± 1.65 mV and − 42.70 ± 1.23 mV, respectively. This reduction in absolute zeta potential is likely attributable to structural rearrangements induced by ultrasonication. Previous studies have shown that ultrasonic treatment can promote the unfolding of secondary structures and increase the exposure of nonpolar hydrophobic residues, which may reduce the abundance of negatively charged groups on the particle surface and consequently decrease the surface charge [41]. Another study similarly reported that high-intensity ultrasonication reduced the negative surface charge of proteins, weakened the electrostatic barrier, and enhanced adsorption at the interface [42]. Notably, SS exhibited a broader decrease in zeta potential than NS, indicating that succinylated protein-based composite particles were more sensitive to ultrasonic treatment. This enhanced susceptibility is likely associated with the altered molecular conformation and the presence of succinyl functional groups introduced during succinylation.

#### 3.2.2 Comparative analysis of particle size and zeta potential of different particles

**Fig. 3:**
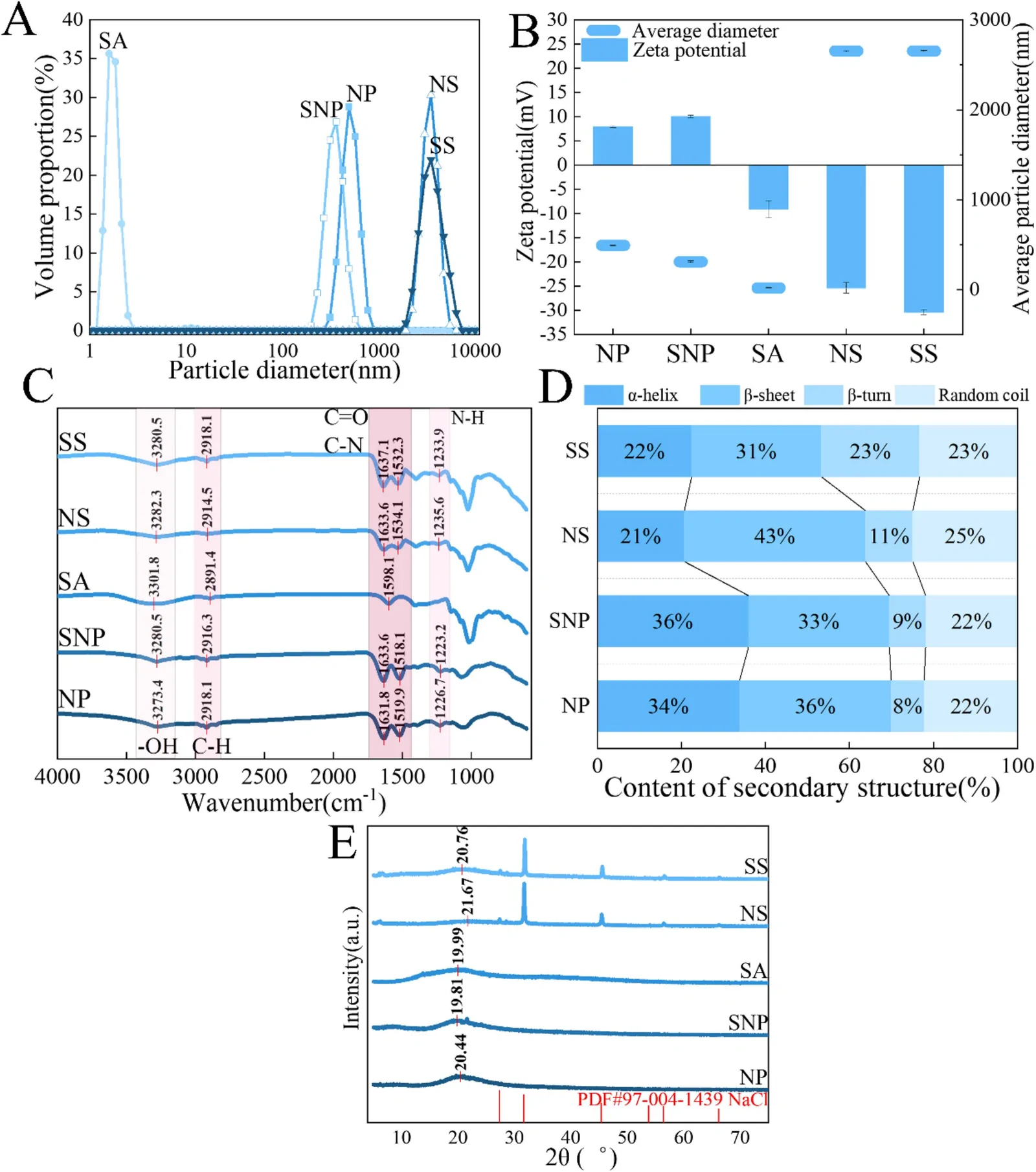
Characterization of Composite Particles.(A) Particle size distribution of the particles. (B) BAverage particle size and zeta potential of the particles. (C) FTIR spectra of different particles. (D) Secondary structure distribution of different particles.(E) XRD spectra of different particles. (F) Conditions for forming stable emulsions. (NP: protein particles, SNP: succinylated protein particles, SA: sodium alginate particles, NS: sodium alginate and protein composite particles, SS: sodium alginate and succinylated protein composite particles).

To investigate the pH range conducive to electrostatic complexation between proteins and polysaccharides, the particle size and zeta potential of proteins and sodium alginate (SA) were measured under various pH conditions. As shown in Fig. S1D, protein particles exhibited a positive charge at pH < 3 and a negative charge at pH > 5. SA was nearly neutral at pH ≈ 1 and negatively charged under all other tested conditions. Based on these observations, positively charged proteins at pH = 1 could form complexes with negatively charged SA at pH = 7 via electrostatic attraction. The modified protein and composite particle displayed a higher negative charge and a smaller particle size (Fig. 3A, Fig. 3B), consistent with previous findings [43]. This enhanced negative charge increased electrostatic repulsion between protein particles, thereby reducing emulsion aggregation. Moreover, the smaller particle size of the succinylated protein facilitated the formation of denser structures with polysaccharides, contributing to the superior stability of succinylated nori protein emulsions (SNPE) compared to their non-succinylated counterparts (NPE). This phenomenon arises from the succinylation-mediated conversion of cationic ε-amino groups to anionic succinamide groups, consequently increasing the negative charge density at the protein surface.

#### 3.2.3 FTIR spectroscopy analysis

FTIR spectroscopy revealed conformational changes in proteins after succinylation. The amide I band consists of several sub-bands corresponding to different secondary structures: α-helix (1664–1648 cm^−1^), β-sheet (1637–1615 cm^−1^ and 1700–1682 cm^−1^), β-turn (1681–1664 cm^−1^), and random coil (1648–1637 cm^−1^). Both interface-adsorbed and non-adsorbed proteins showed redshifts at 1519.9 and 1631.8 cm^−1^ (Fig. 2C), corresponding to amide I (C-N, C-O deformation) and amide II (C-N, N-H stretching) vibrations. A minor shift in the amide III band (N-H, C-N stretching) was also observed. Succinylation induced a blueshift in amide I and redshifts in amides II and III. The narrowed peak at 3500–3200 cm^−1^ indicated N-H to C-N conversion, confirming covalent succinylation. Changes around 3300 cm^−1^ reflect C-H and N-H stretching [44], [45]. These alterations suggest conformational rearrangement, exposing polar groups and altering hydrogen bonding, confirming successful NP succinylation and NP-SA binding.

#### 3.2.4 The secondary structure contents analysis

As shown in Fig. 2D, the secondary structure contents of interface–adsorbed and non–adsorbed proteins were calculated by fitting the amide I band. After succinylation, the β–sheet content of the protein decreased significantly, while the contents of α–helix and β–turn increased. When the protein was combined with polysaccharides, the increase in disordered structures such as β–turn and random coil in the complex compared to the protein alone indicates enhanced conformational flexibility, which underlies the ability of NS and SS to form stable emulsions. Succinylation alters the protein conformation, making it more loose and flexible [46]. Furthermore, existing studies suggest that α–helix is primarily associated with intramolecular structural flexibility, while β–sheet is more related to intermolecular structural stability. Reducing α–helix content and increasing molecular flexibility play a crucial role in enhancing emulsion stability [28]. In addition, the increase in β–sheet and random coil contents may expose more hydrophobic groups, thereby enhancing interfacial activity and improving emulsifying properties. The surface morphology further revealed that succinylation altered the protein secondary structure and contributed to the formation of a denser and more compact core–shell structure by the composite particles at the droplet interface. This is consistent with the microstructure observed by cryo-SEM (Fig. 1B). The balance between the hydrophobic groups introduced by succinylation and the hydrophilic sodium alginate provides important evidence for the subsequent stabilization of the emulsion.

#### 3.2.5 X-ray diffraction analysis

X-ray diffraction (XRD) was employed to examine the crystalline structure of the materials and the intermolecular interactions between components [47]. All samples displayed a broad amorphous halo centered at approximately 2θ = 20°, characteristic of non-crystalline materials. In the crystalline region, NP and NS exhibited diffraction peaks at 2θ = 20.44° and 21.67°, respectively, representing a shift to higher angles relative to SNP (19.81°) and SS (20.76°) (Fig. 2E). This angular shift indicates that succinylation modification and SA incorporation alter the protein's crystal lattice and facilitate new chemical interactions within the protein structure. This further confirms the successful binding between the protein and polysaccharide. The incorporation of SA reduced peak intensity while shifting peaks to higher angles. The elevated diffraction angles observed for NS and SS suggest increased crystallinity, reflecting a more ordered and rigid molecular arrangement, reduced conformational flexibility, and enlarged crystallite size. Additional sharp diffraction peaks detected in NS and SS were identified as NaCl (PDF#97–004-1439), originating from salts introduced during pH adjustment with NaOH and HCl.

### 3.3 Microstructure of Pickering emulsion

#### 3.3.1 Cryo–SEM analysis

High-resolution observation using confocal fluorescence microscopy (CLSM) and cryo–SEM was conducted to examine the distribution of individual particles and composite particles at the interface of Pickering emulsion droplets, thereby assessing the internal oil phase distribution and the structural arrangement of surface particles [48]. From a surface structural perspective, as shown in Fig. 1B, CLSM revealed that aqueous-phase particles aggregated at the oil droplet surface, providing visual evidence for the effective formation of Pickering particles. Cryo–SEM cross–sectional images further demonstrated that the particles formed a core–shell structure that tightly encapsulated the oil droplets. Surface imaging also indicated that succinylation and electrostatic complexation resulted in a denser and more uniform particle shell [49].

#### 3.3.2 CLSM images

**Fig. 4:**
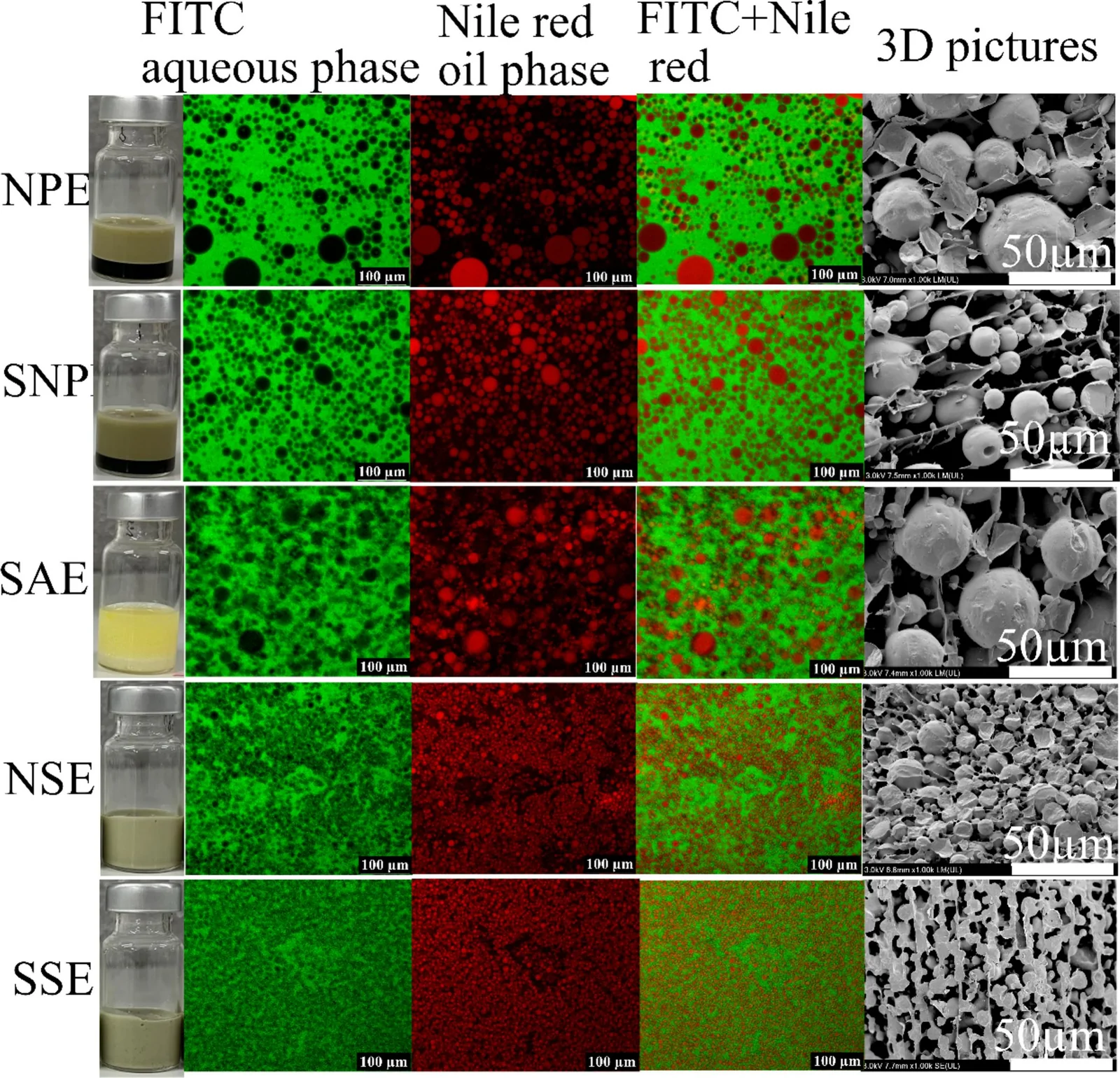
Characterization of emulsions formed by different kinds of particles. (NPE: nori protein particle-stabilized emulsion. SNPE: succinylated nori protein particle-stabilized emulsion. SAE: sodium alginate particle-stabilized emulsion. NSE: sodium alginate and protein composite particle-stabilized emulsion. SSE: sodium alginate and succinylated protein composite particle-stabilized emulsion).

Additionally, the state of the Pickering emulsions after 10 days of storage was documented. NSE and SSE showed minimal phase separation, while NPE and SNPE separated into emulsion and aqueous layers. SAE separated into oil and emulsion layers, a phenomenon attributed to the hydrophobic nature of the protein and the hydrophilic character of the polysaccharide. Similarly, NP and SNP systems showed reduced oil exudation, while SAE is the opposite. The composite system of SA with a small amount of NP/SNP integrated the advantages of both components. Characterization using confocal fluorescence microscopy yielded consistent results. CLSM images clearly showed that SNPE formed smaller and more densely packed oil droplets compared to NPE (Fig. 4). Although SA imparted thickening properties, the resulting emulsion droplets were larger, which may be related to its strong electrostatic repulsion. Similarly, compared to NSE and SSE formed a denser and more stable emulsion, with composite particles producing smaller and more stable droplets [50]. Under cryo–SEM observation at the same 3D magnification (1.00 K × ), SNPE exhibited smaller and more stable oil droplets than NPE, with the surrounding aqueous phase tending to form dense lamellar structures that organized the oil droplets in an orderly arrangement. Evidently, SSE droplets were smaller, denser, and more ordered than those of NSE. This is consistent with the microstructure observed by cryo-SEM. These findings indicate that emulsion droplets formed by binary composite particles perform significantly better than those formed by single particles. This is primarily attributed to the zeta potential of composite particles falling within the range of 30–60 mV and their spatial structure providing sufficient electrostatic repulsion to prevent droplet aggregation. Moreover, the larger particle size of the composites enables more complete and dense encapsulation of the oil droplets [51].

### 3.4 Optimization of parameters affecting Pickering emulsion stability

#### 3.4.1 Characterization of the Pickering emulsion with the different particle contents

To identify the optimal formulation parameters, emulsions were systematically characterized across a range of particle contents, protein-to-polysaccharide ratios, and oil phase fractions). Emulsion activity index (EAI) reflects the ability of an emulsifier to rapidly adsorb at the oil–water interface at the moment of emulsification, whereas emulsion stability index (ESI) represents the capacity of the emulsion to maintain a stable state after formation. The effect of particle concentration on emulsion properties was systematically evaluated (Fig. S2). At low concentrations (0.5%-1%), EAI values were exceptionally high (Fig. S2A), indicating efficient interfacial adsorption. However, the correspondingly low stability precluded this range from optimal formulation. Both EAI and ESI reached maxima at 3% particles, consistent with the enhancing effect of polysaccharides on protein EAI reported by Yao et al. [52]. CI values decreased markedly with increasing particle concentration (Fig. S2B): from 70.38% to 2.89% for SSE and from 71.63% to 1.30% for NSE over the 0.5%-5% range, reflecting progressively improved stability. HIS measurements (Figs. S2C, S2D) revealed time-dependent phase separation at ≤ 2.5% particles, with SSE consistently outperforming NSE. Visual inspection after 10 days (Fig. S2E, S2F) confirmed that emulsions with ≥ 3% particles exhibited no visible phase separation [53]. Accordingly, a particle concentration of ≥ 3% was identified as the optimal formulation parameter.

#### 3.4.2 Characterization of the Pickering emulsion with the different protein-to-polysaccharide ratio

The protein-to-SA ratio critically affected emulsion stability (Fig. S3). The maximum EAI and ESI values observed at the 2:10 ratio suggest that this formulation achieves an optimal balance, providing complex particles with suitable interfacial adsorption and wettability. Deviations from this ratio compromise stability, with excess protein leading to aqueous phase separation and excess polysaccharide promoting oil separation. Unlike many systems using high protein ratios (1.25:1–8:1) [54], our optimum was polysaccharide-rich, likely due to compositional differences [55], [56]. Centrifugal stability (Fig. S3B) and 10-day HIS monitoring (Fig. S3C, S3D) confirmed that higher protein content accelerated phase separation, resulting in higher HIS values and CI values. Ratios 1:11 and 2:10 remained stable. Visual records (Fig. S3E and F) corroborated these findings. Notably, these emulsions used suboptimal particle content (no more than 3%); greater stability is anticipated under optimized conditions.

#### 3.4.3 Characterization of the Pickering emulsion with the different oil phase volume fraction

The effect of oil phase volume fraction (φ) on the formation and stability of Pickering emulsions was systematically investigated over the range of 10%-80% (Fig. S4). Measurements of the emulsion activity index (EAI) and emulsion stability index (ESI) revealed a non-linear relationship between oil content and emulsion performance. As shown in Fig. S4A, both EAI and ESI initially increased with increasing oil phase proportion, reached a maximum at φ = 75%, and then decreased. At this optimal oil fraction, particles were nearly completely adsorbed at the oil–water interface, enabling effective encapsulation and stabilization of newly formed droplets. Deviations from this optimal oil content-whether too low or too high-resulted in phase separation, attributable to an excess or deficiency of particles relative to the interfacial area (Fig. S4E and F). This finding is consistent with previous studies, which reported maximum emulsion stability at an oil fraction of 70% [57]. Similarly, the emulsions exhibited optimal centrifugal stability at φ = 75% (Fig. S4B), suggesting that this oil fraction represents a critical threshold at which an optimal balance is achieved between interfacial particle packing efficiency and overall network structural strength [58]. The stability of emulsions over time was monitored by measuring the height of interface separation (HIS) at different time points (Fig. S4C and D). Emulsions with oil phase fractions ranging from 10% to 60% underwent gradual phase separation over time, with the volume of the separated aqueous phase progressively decreasing. In contrast, Pickering emulsions with oil fractions of 70% and 75% exhibited no phase separation throughout the observation period. As the oil phase proportion increased, the number of oil droplets in the system increased while the average inter-droplet distance decreased, significantly enhancing the frequency of droplet collisions and steric hindrance during upward movement, thereby delaying creaming and coalescence [55]. The above results were fully confirmed by the photographic observations of centrifugal stability (Fig. S5). Whether emulsions could be formed and their long-term stability using different particle contents and protein-to-polysaccharide ratios were evaluated, as illustrated in Fig. 4D. the particle content, protein-to-polysaccharide ratio, and oil phase fraction are key determinants of HIPPE properties.

### 3.5 Interfacial adsorption behavior analysis of HIPPE

#### 3.5.1 The three-phase contact angle of HIPPE

**Fig. 5:**
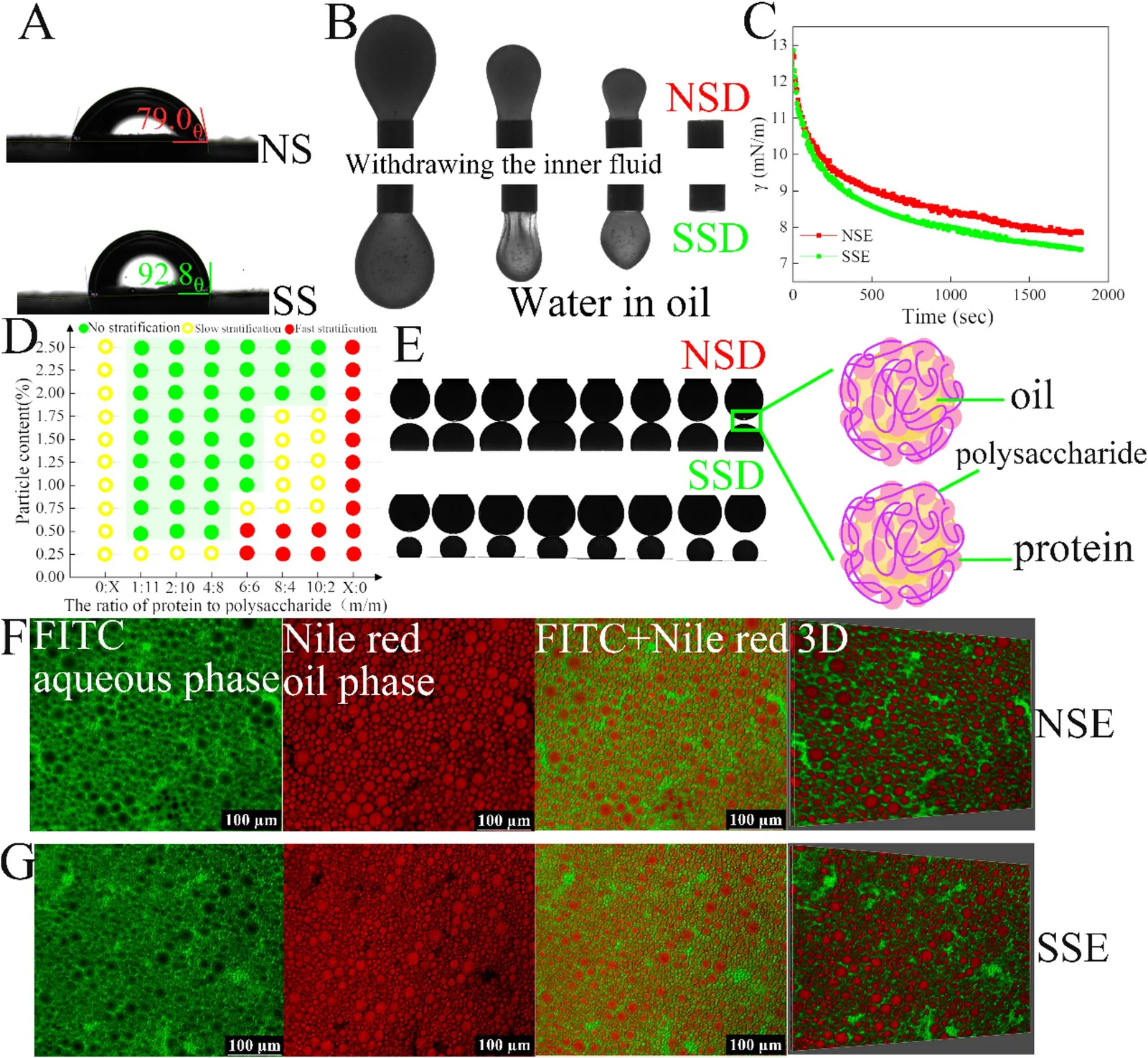
Interfacial adsorption behavior and stability evaluation of high internal phase Pickering emulsions (HIPPE).(A) Three-phase contact angles (*θ*) of NS and SS. (B) Two sequences of snapshots showing the interfacial jamming and buckling when withdrawing the inner fluid due to the electrostatic complexations at the interface.(C) Dynamic interfacial tension of of oil-in-water droplets (D) NS- and SS-stabilized water droplets are stable against coalescence, compression, and deformation., (E)CLSM of NSE. (F) CLSM of NSE. (NS: sodium alginate and native protein composite particles. SS: sodium alginate and succinylated protein composite particles. NSD:water drop of NS. SSD: water drop of NS. NSE: sodium alginate and protein composite particle-stabilized high internal phase Pickering emulsions. SSE: sodium alginate and succinylated protein composite particle-stabilized high internal phase Pickering emulsions).

The physicochemical properties and structural modifications of composite particles significantly influence their interfacial behavior, including interfacial tension and adhesion. The three-phase contact angle (θ) determines particle behavior at the oil–water interface: θ ≫ 90° favors W/O emulsions, θ ≪ 90° favors O/W emulsions, and θ near 90° maximizes amphiphilicity and stability [59]. The contact angle of NS ranged from 76.3° to 79°, while that of the succinylated SS ranged from 92.8° to 94°, placing it closer to 90° (Fig. 5A). This discovery is consistent with previous findings that the combination of hydrophobic proteins and hydrophilic polysaccharides enhances amphiphilicity. For comparison, previous studies have reported a three-phase contact angle of 39.8 ± 9.8° for monosaccharides, reflecting their high hydrophilicity. The complexation of hydrophobic proteins with hydrophilic sodium alginate imparts amphiphilic wettability to the resulting particles, promoting Pickering emulsion stabilization. Consistent with Zhu, et al. reported that treatments such as heating, ultrasonication, and pH adjustment can expose hydrophobic structures in particles, thereby enhancing their wettability [60].

#### 3.5.2 Attractive interactions of HIPPE

To simulate the microscopic morphology of particle-stabilized interfaces, an aqueous phase containing composite particles was dispensed into the oil phase without detaching from the probe. The interface formed between the water droplet and the surrounding oil phase was used to model the interfacial behavior of solid particles. A higher degree of droplet sphericity indicates better prevention of coalescence and adhesion between the internal and external oil phases, thereby enhancing stability. During inward retraction, droplets that remained intact and did not spread upon detachment from the interface exhibited superior stability (Fig. 5B) [31].Similarly, when a water droplet stabilized by NS or SS was brought into proximity with another NS/SS-stabilized droplet, subjected to compression, and then separated, the droplets demonstrated resistance to coalescence, compression, and deformation throughout the process (Fig. 5E). This finding was further supported by the subsequent texture and rheology results. Notably, no mutual attraction was observed between the droplets upon separation, indicating the formation of a stable shell structure by the particles. Even upon contact, the droplets rebounded like rubber balls without adhering or coalescing, further confirming the robustness of the interfacial film.

#### 3.5.3 Interfacial property of HIPPE

The dynamic adsorption behavior of composite particles at the oil–water interface was evaluated through interfacial tension measurements (Fig. 5C). The interfacial tension of NSE decreased to 10.5 mN∙m-^1^ within 160 s, while that of SSE decreased more rapidly to 10 mN∙m^−1^ within 125 s. This accelerated adsorption kinetics for SSE reflects the higher mobility of succinylated protein polymers, enabling faster adhesion to the interface via hydrophobic interactions during the initial stage. Moreover, the consistently lower interfacial tension of SSE during the subsequent phase indicates enhanced dispersibility and more effective interfacial coverage, providing both kinetic and thermodynamic advantages for interface stabilization [61]. These observations align with reports that protein-polysaccharide extracts from brown algae enhance emulsification by forming viscoelastic interfacial films and reducing surface tension [20]. CLSM images of high internal phase Pickering emulsions (Fig. 5F and G) revealed that SSE-stabilized droplets were smaller than their NSE-stabilized counterparts. This morphological difference corroborates the superior contact angle, interfacial tension, and anti-adhesion properties demonstrated by the succinylated composite particles.

### 3.6 Rheological analysis of HIPPE

#### 3.6.1 The strain sweep test analysis

**Fig. 6:**
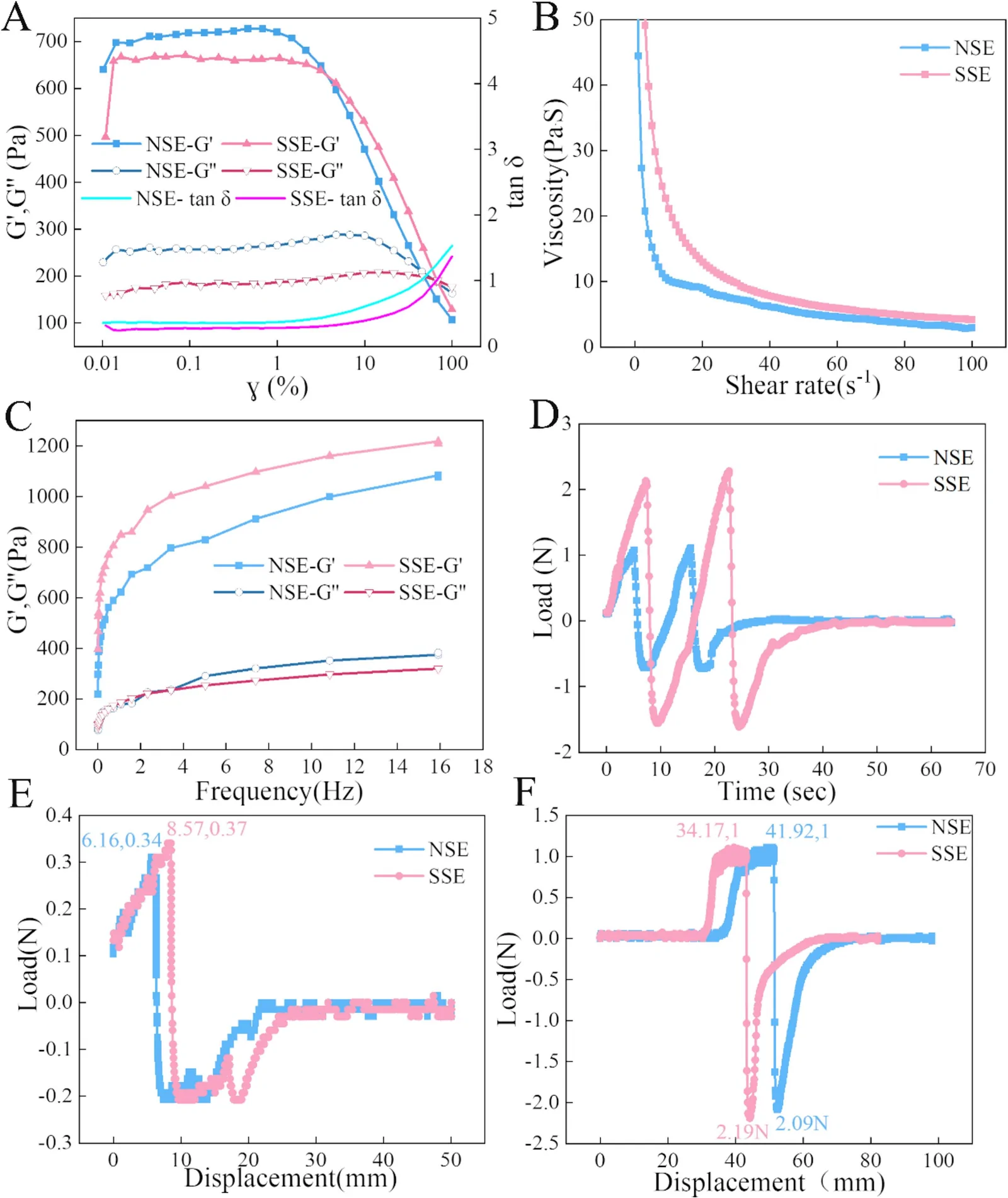
Effect of different Pickering particles on rheological properties of HIPPE. (A) Strain scanning curve, (B) Apparent viscosity-shear rate curve (C) Storage modulus, loss modulus- frequency curve. (D) Texture profile analysis (TPA). (E) Shear stress curves. (F) Creep recovery curves. (NSE: sodium alginate and protein composite particle-stabilized high internal phase Pickering emulsions. SSE: sodium alginate and succinylated protein composite particle-stabilized high internal phase Pickering emulsions).

The linear viscoelastic region (LVR) of the HIPPE was determined using the strain sweep test, in which the strain increased from 0.01% to 100% at 1.592 Hz. As shown in Fig. 6A, when the strain is less than 4%, G′ and G″ remain constant, indicating that the emulsion is within the linear viscoelastic region (LVR). At this stage, tan δ (G″/G′) < 1 indicates that solid-like behavior dominates and SSE exhibits superior strain tolerance and deformation resistance (46.7%) and significantly outperforms NSE (31.6%). As the strain gradually increases to 100%, the storage modulus (G′) of all emulsions decreases significantly. This indicates the gradual weakening of the gel-like structure in HIPPE, leading to the liquefaction of the samples. Such a phase transition suggests that the high strain disrupts the interfacial particle network, making viscous dissipation the dominant mechanism [62]. The maximum strain that high internal phase emulsion (HIPPE) samples can withstand without causing permanent structural damage is referred to as the critical strain. The composite particles formed by modified proteins significantly increase the critical strain, indicating that the droplets are more densely packed and form a stronger, more deformation-resistant gel-like structure. This finding is consistent with the results shown in Fig. 5F and G.

#### 3.6.2 Steady shear test analysis

The shear-dependent rheological properties of emulsions fundamentally reflect the interactions between droplets. As shown in Fig. 6B, the apparent viscosity decreases as the shear rate increases from 0.1 s^−1^ to 100 s^−1^, with both emulsions exhibiting characteristic pseudoplastic shear-thinning behavior [41]. Emulsions containing modified proteins exhibit higher viscosity. Generally, increased viscosity helps inhibit particle flotation and sedimentation, thereby suppressing phase separation and enhancing emulsion stability [63]. Based on the curve fitting with the Casson model τ=^n^√(τ_0_^n^+(ɣ̇ηᵨ)^n^, the yield stress τ_0_ for NSE is 76.89 Pa, while for SSE it is 137.3 Pa. Physically, this indicates that a “starting stress” of approximately τ_0_ must be overcome for the material to transition from a static state to flow, providing a comprehensive rheological description of its behavior. This finding is consistent with the subsequent creep test results.

#### 3.6.3 Frequency sweep test analysis

Typically, the storage modulus (G′) and loss modulus (G″) are used to evaluate the elastic and viscous properties of emulsions, respectively. As shown in Fig. 6C, both G′ and G″ increase with frequency, exhibiting significant frequency dependence. This behavior is characteristic of weak gel networks, indicating that the gel-like structure of the emulsion is primarily stabilized by non-covalent interactions. The frequency sweep results of NSE and SSE indicate that within the range of 0.1 to 16 Hz, G′ consistently exceeds G″, demonstrating that the emulsions exhibit gel-like elastic behavior within the linear viscoelastic region [64]. These observations highlight that the SSE possesses a more ordered structure and a more efficient elastic network, wherein elastic behavior predominates.

#### 3.6.4 TPA, creep and creep recovery analysis

In this study, the texture properties of the HIPPE were explored using the TPA method. The parameters (including hardness, viscosity, cohesiveness and chewiness) were used to evaluate the application feasibility of the HIPPE in food system. The obtained results are shown in Fig. 6D and table S1. The hardness, viscosity, cohesiveness, springiness, and chewiness of the HIPPE progressively increased upon protein modification [65]. During stress testing, it can be observed that to achieve the same strain of 50%, the hardness of NSE is 0.34 ± 0.00 N, while that of SSE is 0.37 ± 0.06 N, as shown in Fig. 6E. The corresponding compression displacement at maximum hardness is also the greatest for SSE. SSE requires greater force and displacement to achieve deformation, indicating its stronger resistance to deformation and higher rigidity. In creep testing (Fig. 6F), to reach the maximum stress of 1 N, NSE requires greater displacement and longer time than SSE, suggesting that it encounters less resistance and flows more easily. In contrast, SSE attained the maximum stress of 1 N within a shorter displacement, suggesting a more compact internal architecture and superior resistance to structural disruption. During the creep recovery phase, SSE requires a greater force of 2.19 ± 0.23 N (compared to 2.09 ± 0.17 N for NSE) to return to its original state. These findings collectively indicate that SSE possesses a more stable structure than NSE.

### 3.7 The temperature stability analysis of HIPPE

Temperature critically influences emulsion behavior by modulating polysaccharide structure and functional group interactions, thereby affecting emulsifying capacity and stability. At pH 7, the emulsions maintained stability at both 4 °C (NSE: 24.62 ± 0.02 µm, SSE: 22.27 ± 0.08 µm) and 25 °C (NSE: 24.70 ± 0.03 µm, SSE: 23.08 ± 0.09 µm) characterized by small and uniformly distributed droplet sizes. This is consistent with the previous findings regarding the droplet microstructure. As the temperature increased, the droplet size of the emulsions progressively enlarged. At 100 °C, droplet aggregation was evident, and visual inspection revealed pronounced oil separation (Fig. 6A). This destabilization is attributed to the breakdown of hydrogen bonds and polysaccharide cross-links, causing partial gel melting, protein denaturation and intensify Brownian motion and loss of structural integrity [49], [66]. Succinylated particle-stabilized emulsions (SSE) consistently exhibited smaller droplet sizes than unmodified NSE, demonstrating enhanced thermal stability, in agreement with Lian et al. [28]. Freezing at −20 °C resulted in irreversible demulsification, with extensive oil separation (Fig. S6). SSE showed superior freeze–thaw resistance compared to NSE, as evidenced by smaller droplet sizes and reduced oil separation. This improvement is attributed to the protective effect of modified proteins against ice crystal-induced damage to the interfacial layer during freezing and thawing [28], [67]. Enhancing freeze–thaw stability remains a key challenge for the practical application of HIPPE in food transportation and storage.

Except under freezing conditions, the zeta potential of the emulsions at other temperatures ranged from the range −24.8 mV to −58.9 mV, which is a sutible range for the Pickering emulsion formation (Fig. 6C). Notably, across all tested temperatures, the absolute zeta potential of SSE stabilized by modified protein composite particles was consistently higher than that of NSE. This enhancement is primarily attributed to succinylation, which covalently attaches negatively charged succinyl groups to the ε-NH_3_^+^ groups of lysine residues, thereby increasing the net negative charge of the protein [28]. Furthermore, a notable transition was observed starting from 50 °C, where both the slope of the mean square displacement (MSD) curve and the radius of gyration increased significantly [68]. This behavior is primarily attributed to temperature-induced changes in the fluidity and hydrophobicity of the emulsifier, ultimately leading to phase separation between the aqueous and oil phases. As can be observed, it is adverse for the store in −20 °C and exceed 50 °C.

### 3.8 pH stability analysis of HIPPE

**Fig. 7:**
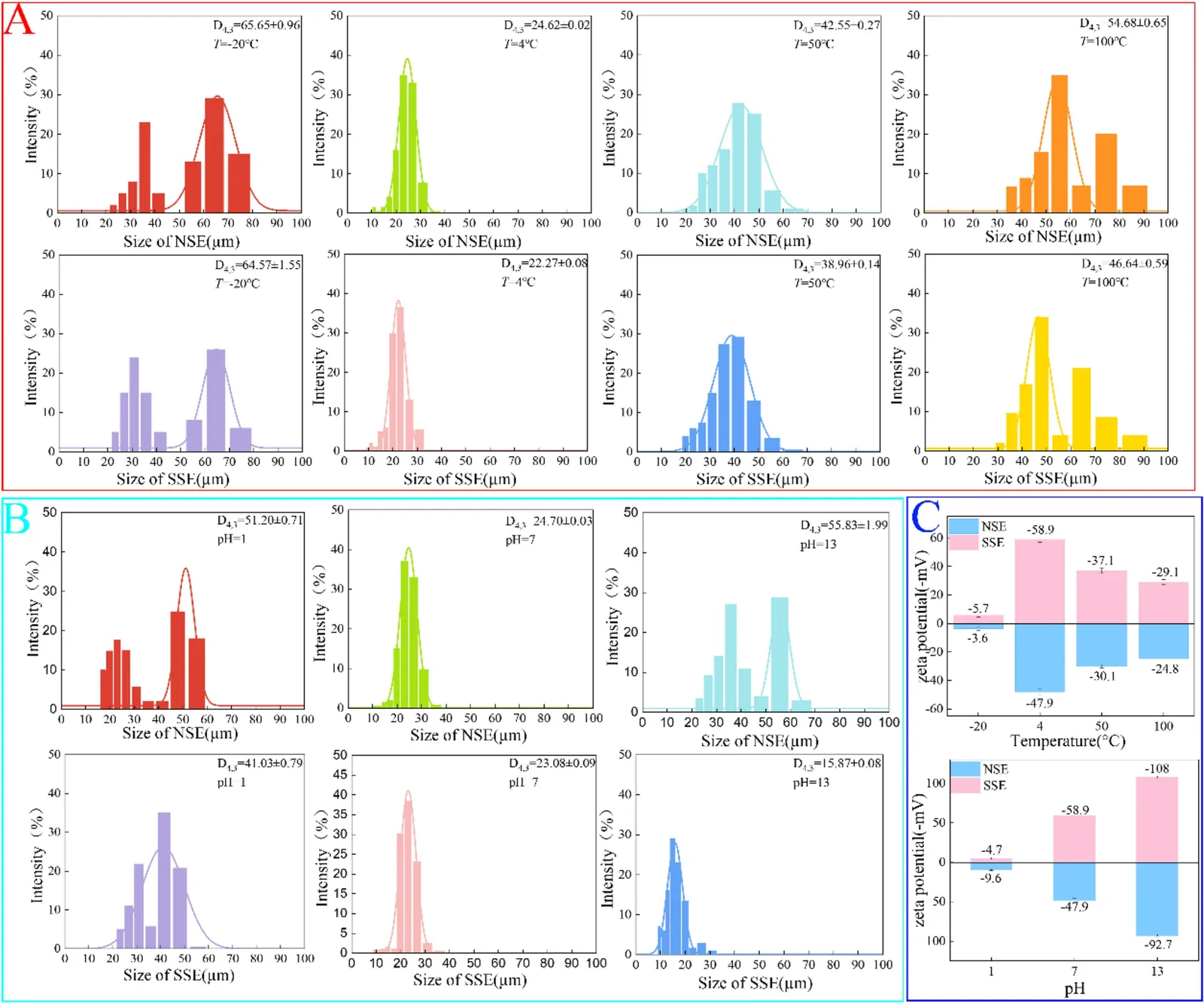
Ph and temperature responsiveness of emulsions. **(A)** Particle size distribution of NSE and SSE at different pH values. **(B)** Particle size distribution of NSE and SSE at different temperatures. **(C)** Zeta potential of NSE and SSE at different temperatures and different pH values (NSE: sodium alginate and protein composite particle-stabilized high internal phase Pickering emulsions. SSE: sodium alginate and succinylated protein composite particle-stabilized high internal phase Pickering emulsions).

pH-responsive analysis reveals that the stability, microstructure, and functional release behavior of high internal phase Pickering emulsions (HIPPE) can be precisely modulated by changes in environmental pH. This tunability provides a critical foundation for their design and application as intelligent responsive materials. Fig. 7 presents optical microscopy images, particle size distributions, visual appearance, and zeta potential measurements of HIPPE under acidic (pH = 1), neutral (pH = 7), and alkaline (pH = 13) conditions. At pH = 1, protonation of the carboxyl groups of sodium alginate induces gel-like aggregation. Internal oil droplets become embedded within the gel matrix, while external oil separates, resulting in a clumpy, non-flowing emulsion. Optical microscopy reveals that the oil droplets within these clumps are smaller and unevenly distributed. Under neutral conditions, the HIPPE exhibit optimal stability, characterized by a uniform droplet size distribution (20–30 µm). Notably, SSE displays smaller droplet sizes than NSE, indicating superior emulsion stability. Under alkaline conditions, the emulsion undergoes demulsification and becomes flowable. NSE exhibits visible oil separation, whereas SSE remains unstratified with finely dispersed oil droplets. This difference may be attributed to the increased solubility of the modified protein under alkaline conditions, which enables the particles to form a denser interfacial layer [69].

It is generally accepted that when the zeta potential of solid particles exceeds 30 mV, the electrostatic repulsion generated by the high surface charge contributes significantly to the overall stability of the colloidal system [48], [70]. In the Fig. 7C, low zeta potential of pH = 1 caused phase separation and sodium alginate precipitation. At pH = 7, zeta potential increased, enhancing repulsion and stability among the particles. The study found the lagest zeta potential of the stable emulsion can reach −75 mV [20], [71]. At pH = 13, the extremely high zeta potential generated excessive interparticle repulsion, leading to emulsion rupture and the onset of flowability. This behavior is supported by all-atom molecular dynamics simulations, which showed an increased slope in the average azimuth shift curve and changes in the radius of gyration, indicating initial emulsifier aggregation followed by migration into the aqueous phase as pH decreased, ultimately causing demulsification [68], [72]. Interestingly, we observed that by adjusting the emulsion to an alkaline pH and subsequently applying vortex oscillation, the emulsion could undergo a reversible process from demulsification to re-emulsification. The pH responsiveness of HIPPE enables their application as smart materials for the delivery of bioactive macromolecules, the control of reaction switching, and the recovery of enzymes through demulsification and re-emulsification.

### 3.9 Properties of high-internal-phase Pickering emulsions (HIPPE) gels

#### 3.9.1 Microstructure and strength of HIPPE gels

**Fig. 8:**
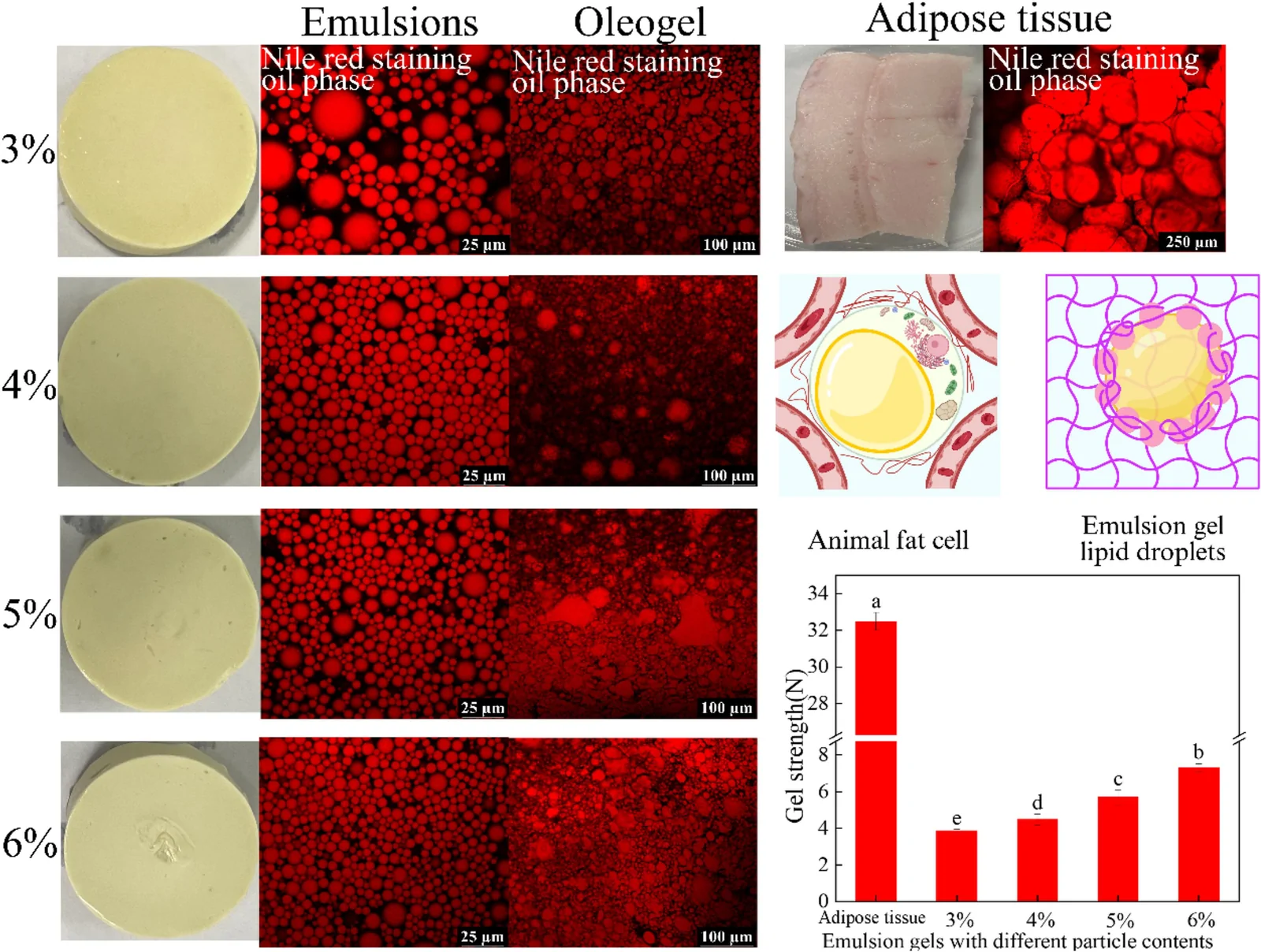
CLSM of HIPPE and HIPPE gels and gel strength under different particle contents.

Fig. 8 presents photographic and confocal laser scanning microscopy (CLSM) images of Pickering emulsion gels prepared with varying particle contents. As the particle concentration increased, the oil droplets became smaller and more densely packed, with optimal uniformity and density observed at particle contents between 4% and 5%. In these emulsion gels, calcium ions cross-link with sodium alginate in the continuous phase, forming a dense network framework.When the particle content exceeded 5%, droplet aggregation became evident. The gel strength progressively increased with rising particle content, attributable to the enhanced density and mechanical strength of the network structure resulting from the increased concentration of sodium alginate polymer in the continuous phase [73]. This denser network reduces the available space for oil droplets, rendering them more susceptible to compression and collision, ultimately leading to the aggregation of larger droplets. This phenomenon explains the destabilization of emulsion gels when the particle content surpasses a critical threshold. Regrettably, compared with plant-based alternatives, Pork fat tissue exhibits superior gel strength, highlighting the need for further in-depth research on plant-based fats to overcome the practical challenges in this area.

#### 3.9.2 Freeze-thaw stability and thermal behavior of HIPPE gels

**Fig. 9:**
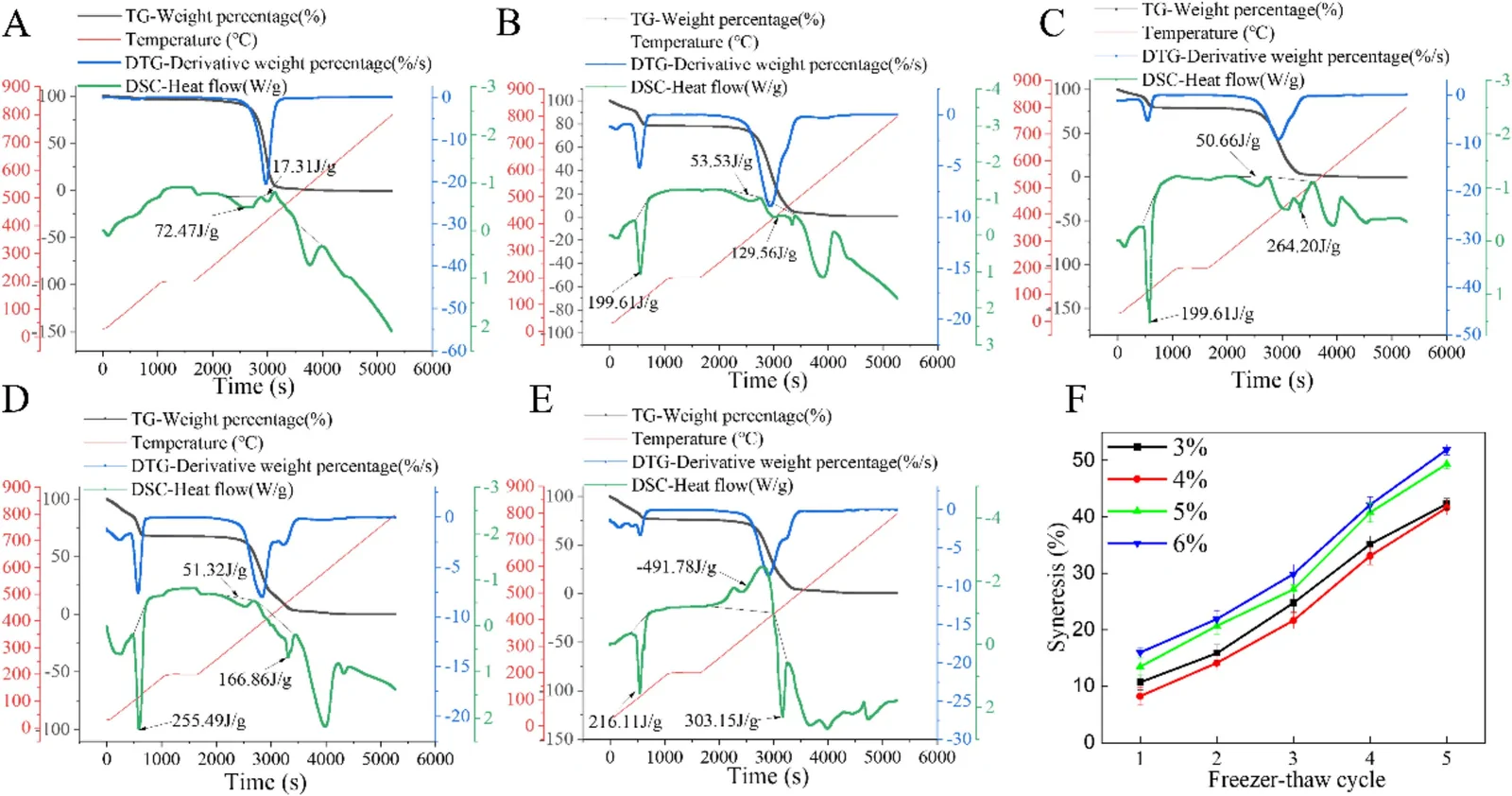
DSC-TG thermograms and Freeze-thaw stability of porcine adipose tissue and the HIPPE gels. (A) DSC-TG thermograms of 3% particle content. (B) DSC-TG thermograms of 4% particle content. (C) DSC-TG thermograms of 5% particle content. (D) DSC-TG thermograms of 6% particle content. (E) freeze–thaw stability of the HIPPE gels.

Furthermore, the freeze–thaw stability of the emulsion gels was rigorously evaluated. During freeze–thaw cycling, thermal energy fluctuations and water phase transitions destabilize the gel network, inducing syneresis (dehydration shrinkage). As the number of freeze–thaw cycles increased, the syneresis rate of all samples progressively increased. This trend can be attributed to the physical disruption inflicted upon the gel network by ice crystal formation and growth, as evidenced in Fig. 9F. Notably, the 4% particle concentration sample consistently exhibited the best freeze–thaw stability across all cycles. This superior stability is likely attributable to its highly dense gel network and the enhanced stability of the encapsulated emulsion oil droplets, the finding consistent with the observed superior microstructural integrity. In contrast, the freeze–thaw stability of samples with higher particle concentrations (> 4%) progressively diminished with increasing cycles. This reduction in stability is attributed to increased aggregation of larger gel structures and oil droplets within the hybrid network, a phenomenon aligned with our microstructural stability observations (Fig. 8). These results collectively demonstrate that particle concentration significantly influences the stability of Pickering emulsion gels under freeze–thaw stress conditions [50].

To investigate the physical characteristics of HIPPE gels in mimicking animal fat during cooking, the thermal behavior of porcine adipose tissue and HIPPE gels with varying particle contents was analyzed using simultaneous thermogravimetry–differential scanning calorimetry (TG-DSC). The thermal decomposition curves (DSC), weight loss profiles (TG), and derivative thermogravimetry (DTG) are presented in Fig. 9. Two distinct temperature regions were observed in the TG curves. In the first region, the TG curve of porcine adipose tissue showed only a minimal change, with a remaining weight percentage of 90.54%, reflecting its extremely low free water content. In contrast, an initial upward peak around 100 °C in the HIPPE gels (3%-6%) corresponded to water evaporation, with the remaining weight percentages being 73.62%, 73.82%, 63.07%, and 72.12%, respectively. This is related to the water that has not been completely removed from the gel surface. All samples exhibited a second weight loss and absorption peak event between 380 °C and 481 °C, which was attributed to the thermal decomposition of oil and protein components.The enthalpy change (ΔH) showed a nonlinear but overall increasing trend with particle content: 17.31 J/g for pork fat tissue, 129.56 J/g for 3%, 264.20 J/g for 4%, 166.86 J/g for 5%, and -491.78 J/g for 6%. A higher enthalpy value generally indicates a denser internal molecular structure, and the 4% particle content exhibited a relatively high ΔH value. In contrast, the abnormal exothermic enthalpy observed at 6% suggests that, after dehydration, the high concentration of Pickering particles formed a dense organic–inorganic composite network. Due to hindered heat and mass transfer, interfacial catalytic effects, and spatial confinement within the excessive three-dimensional particle network, the net thermal effect in the high-temperature region shifted from endothermic to exothermic. Above 500 °C, the sample weight remained stable with no further changes. Collectively, these results demonstrate that SNP and SA significantly influence the thermal behavior of HIPPE gels, and formulations with lower particle content show significant potential as animal fat analogues.

### 3.10 Properties of meat patties with the fat analogues

#### 3.10.1 Appearance and color analysis

**Fig. 10:**
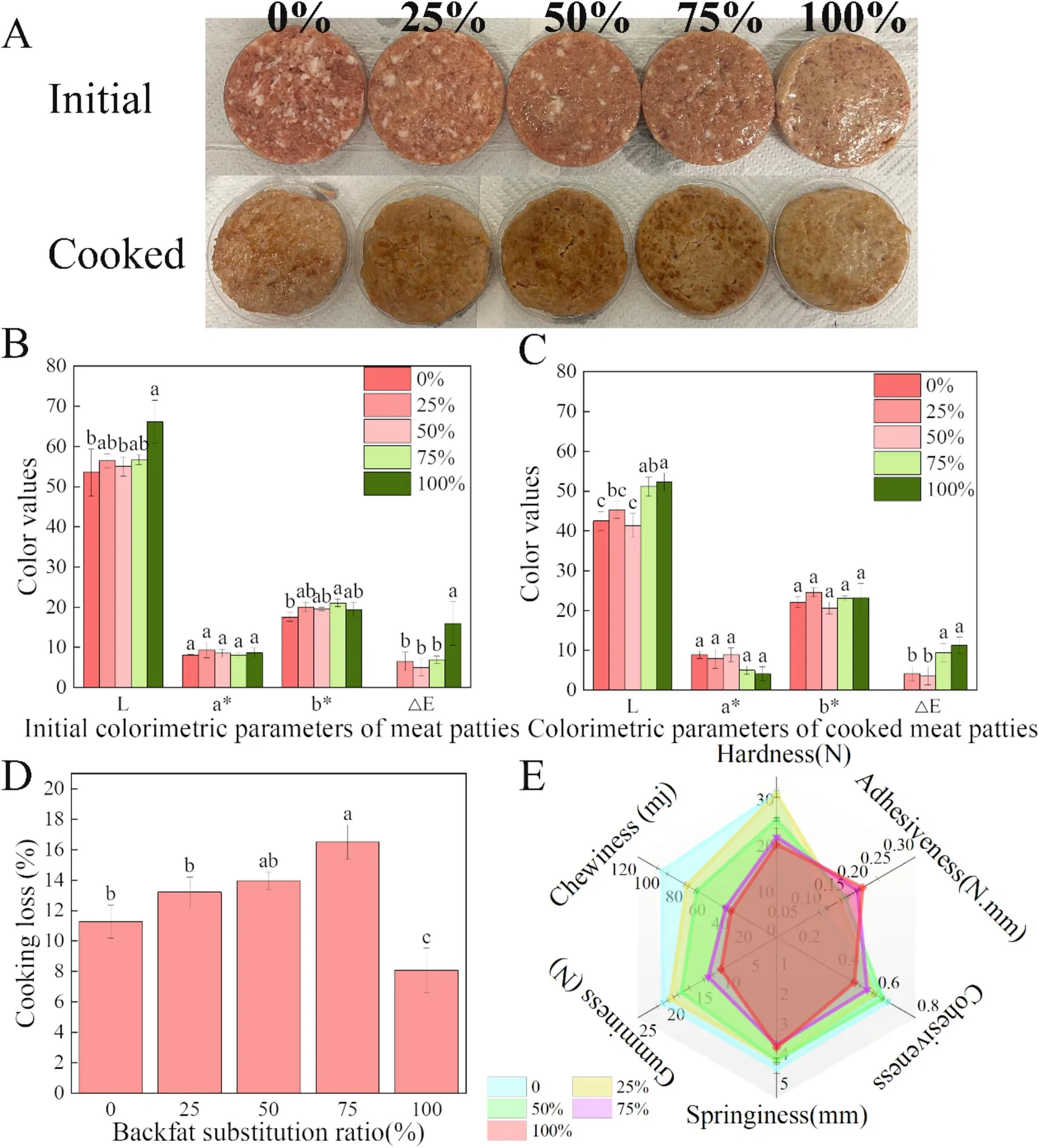
Physicochemical and textural properties of meat patties with different content of fat analogues before and after cooking. (A) photographs of meat patties before and after cooking. (B) color parameters of meat patties before cooking. (C) color parameters of meat patties after cooking. (D) cooking loss of meat patties. (E) texture profile of meat patties.

Appearance and colour are important quality attributes of meat products and strongly influence consumer preference. Fig. 10A and B show the appearance of patties with different levels of fat analogues before and after cooking. It was evident that the incorporation of the fat analogues altered both the colour and texture of the patties. Compared with the control patties without fat analogues, the inherent yellow-green colour of the fat analogues partially masked the typical colour contrast derived from backfat and lean meat. From a visual perspective, slight surface cracks were observed in the cooked patties at 25%, 50%, and 75% replacement levels, whereas the patties with 100% fat replacement exhibited a texture more comparable to that of the control sample. This phenomenon may be attributed to the relatively low proportion of HIPPE gels in the partially substituted formulations. After incorporation into the lean meat matrix, the amount of HIPPE gels at these replacement levels may have been insufficient to provide the rigid interfacial structure and structural stability required for cohesive patties formation. As a result, the lean meat particles were less able to aggregate into a compact and integrated matrix than in the 100% replacement system. These results suggest that the fat-to-lean ratio plays a critical role in determining the appearance and textural properties of the patties. Moreover, the structural contribution of the high internal phase Pickering emulsion became increasingly evident as the level of fat replacement increased [37].

The formulation of the patties significantly affected their colour parameters, including lightness (L), redness (a), and yellowness (b*). Fig. 10C presents the colour measurements of emulsified meat patties prepared with different levels of fat replacement. The incorporation of the fat analogue significantly increased the L* value from 53.54 ± 5.85 to 66.18 ± 5.35, indicating enhanced lightness. The a* value also increased from 8.12 ± 0.10 to 8.70 ± 0.15, reflecting an increase in redness. Similarly, the b* value rose from 17.56 ± 1.07 to 19.44 ± 1.69, reflecting an increase in redness.yellowness. These changes may be partially attributed to the intrinsic colour of flaxseed oil and nori protein in the fat analogue, which could intensify the overall appearance of the patties[74]. In addition, the enhancement in colour may be related to the formation of emulsions between vegetable oil, muscle proteins, and water in the low-fat formulations, which alters light scattering and thereby increases the overall gloss and brightness of the product [37]. Previous studies have also suggested that gels can influence product colour either through their own pigments or through interactions with other components in emulsified meat systems, thereby promoting colour development. After cooking, the a value decreased with increasing fat replacement level, whereas the L and b* values increased. This trend is consistent with the findings reported by Zhuang et al. for low-fat meat batters [36]. Moreover, colour changes in cooked patties may also be associated with caramelisation and Maillard reactions occurring during thermal processing, both of which can further influence the final appearance of the product.

#### 3.10.2 Cooking loss analysis

The stable retention of water and lipids in the meat matrix after cooking is a critical quality parameter for emulsified meat products [75]. As the fat replacement level increased(25%-75%), the cooking loss of the experimental groups gradually increased (Fig. 10D). Notably, no significant differences were found between the 25% and 50% replacement groups and the control (*P* > 0.05). The increase in cooking loss at higher replacement levels may be attributed to the greater incorporation of high-internal-phase Pickering emulsion gels. During thermal processing, elevated temperatures may disrupt the rigid interfacial structure and stability of the Pickering emulsion gel, resulting in the release of entrapped liquid oil. Consequently, more oil phase migrated to the sample surface and exuded from the patties than in the control, thereby increasing cooking loss. This trend differs from that reported for direct fat replacement using soy protein isolate-based composite gels [37]. The discrepancy may be related to the relatively low oil content of that gel system (12%), in which the high levels of soy protein and oxidized chitin nanowhiskers contributed more effectively to strengthening the meat batter structure. Similar increases in cooking loss with increasing fat replacer addition have also been reported in meat sausages [75], [76]. In the present study, the increased physical separation of the high-internal-phase Pickering emulsion gel by the myofibrillar protein matrix may also have reduced gel continuity and increased porosity, thereby promoting water and oil release during cooking (*P* < 0.05). This effect may further explain the significant deterioration in textural properties observed after cooking. Interestingly, the 100% replacement group exhibited the lowest cooking loss and the smallest porosity, which was closely associated with its more integrated appearance and structural morphology after cooking.

#### 3.10.3 TPA analysis

Fig. 10E and Table 1 shows the textural properties of meat patties with different fat replacement levels. Compared with the control, the hardness decreased significantly from 31.23 ± 0.74 g to 20.31 ± 0.86 g as the fat replacement level increased (*P* < 0.05). This reduction may be attributed to the fact that the composite particle-stabilized HIPPEgels exhibited a softer texture than pork backfat. Previous studies have similarly reported that partial or complete replacement of animal fat with plant-based fat alternatives may negatively affect the texture of meat products. It was also observed that meat patties with 25% and 50% fat replacement showed chewiness, cohesiveness, and springiness values close to those of the control, whereas the 75% and 100% replacement groups exhibited less desirable textural properties (*P* < 0.05). This trend is consistent with the findings of Ma et al. [77]. Likewise, Nacak et al. reported that increasing the proportion of emulsion gel led to decreases in cohesiveness, chewiness, and adhesiveness, which was mainly associated with the higher water content of the gel system [78]. In the present study, the emulsion gel contained more free water than animal fat, as also indicated by the above TG-DSC analysis. From a practical perspective, the reduction in hardness may be advantageous for elderly consumers and individuals requiring foods with improved chewability. Nevertheless, these results suggest that although HIPPE gels shows strong potential as a fat analogue, excessive replacement levels may impair patties quality because of the particle-filling effect and the altered water/fat balance within the meat matrix. Therefore, an appropriate fat replacement level should preferably not exceed 50%.

**Table 1:** Textural analysis profile of meat patties with different levels of fat analogues (0%, 25%, 50%, 75% and 100%).

Result Info.	0%	25%	50%	75%	100%
Hardness(N)	31.46 ± 1.01^a^	31.23 ± 0.74^a^	25.71 ± 0.30^b^	21.89 ± 1.83^c^	20.31 ± 0.86^c^
Adhesiveness(N.mm)	0.09 ± 0.01^b^	0.13 ± 0.02^ab^	0.14 ± 0.01^ab^	0.17 ± 0.00^a^	0.18 ± 0.03^a^
Cohesiveness	0.66 ± 0.00^a^	0.59 ± 0.01^ac^	0.63 ± 0.00^ab^	0.55 ± 0.03^c^	0.49 + 0.01^d^
Springiness(mm)	4.52 ± 0.14^a^	4.23 ± 0.07^a^	4.24 ± 0.06^ad^	3.72 ± 0.15^b^	3.80 ± 0.19^b^
Gumminess(N)	20.53 ± 0.93^a^	19.14 ± 1.35^ab^	17.15 ± 1.43^b^	12.38 ± 0.82^c^	10.07 ± 0.26^c^
Chewiness(mj)	98.87 ± 4.40^a^	77.77 ± 1.70^b^	69.21 ± 0.95^c^	44.21 ± 1.78^d^	39.44 ± 1.09^e^

All data represent the mean of triplicates. Different letters in the same column represent significant differences (*P* < 0.05).

#### 3.10.4 Sensorial analysis

**Fig. 11:**
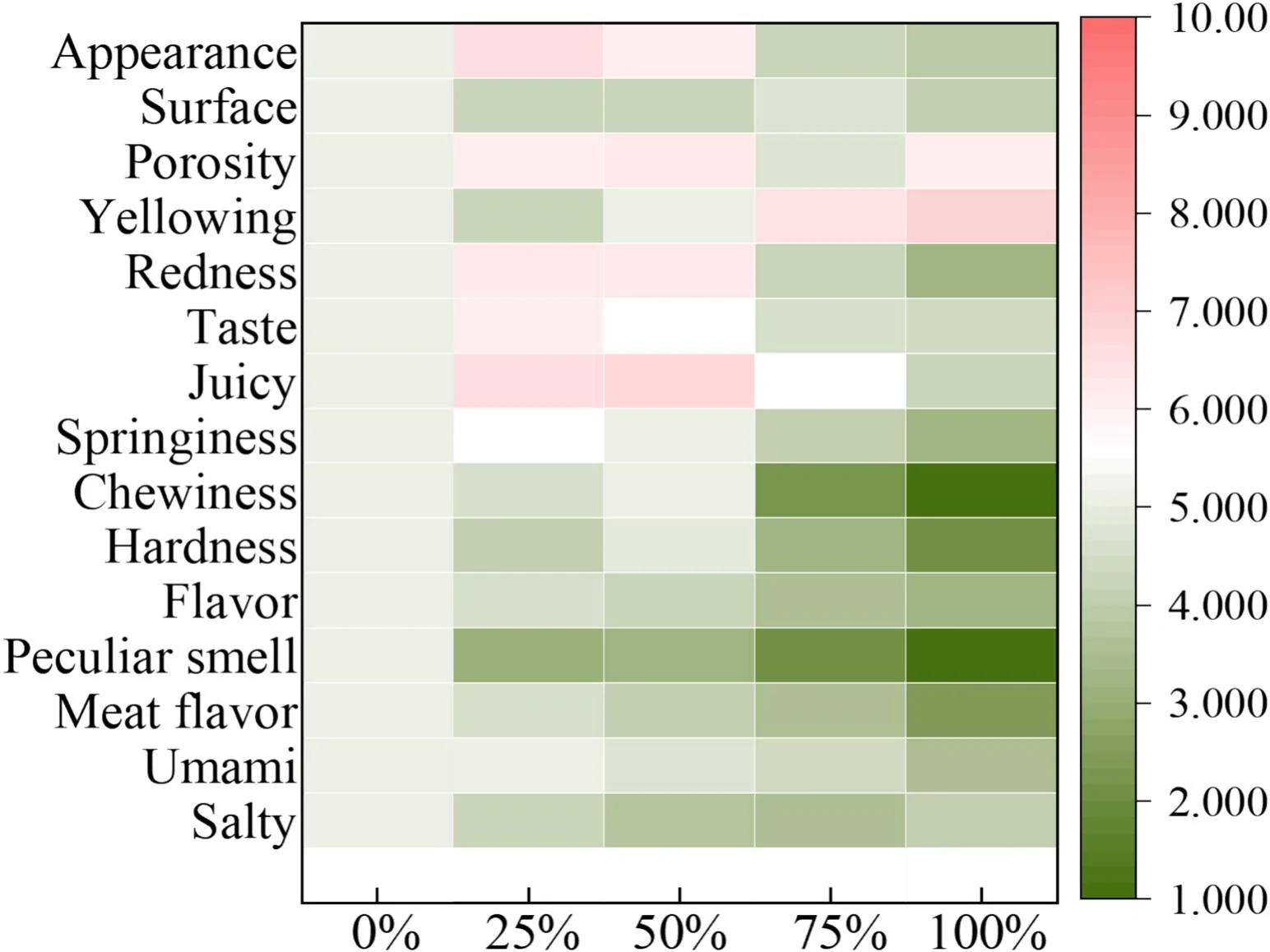
Represented sensory evaluation of meat patties with different content of fat analogues.

Sensory evaluation directly reflects the overall quality of a product and its consumer acceptability, and is considered one of the most intuitive indicators for assessing food quality and consumer preference [79]. As shown in Fig. 11, meat patties in which pork backfat was partially replaced with SS-stabilized HIPPE gels achieved higher sensory scores for springiness, juiciness, and taste at the 25% and 50% replacement levels than the control prepared with pork backfat. However, higher replacement levels led to lower scores for saltiness, umami, and meaty flavour, which may be associated with the development of a peculiar odour. These changes may also be related to the higher cooking loss, reduced hardness, and increased porosity observed at elevated replacement ratios. Although the incorporation of fat replacers slightly decreased the scores for mouthfeel and appearance, no significant differences in flavour scores were found between the 25% and 50% replacement groups and the full-fat control group (*P* > 0.05). Therefore, the HIPPE gels exerted only a limited effect on the flavour of the emulsified meat patties, which is consistent with previous findings [77]. Overall, at fat replacement levels of 25% and 50%, the experimental groups produced sensory qualities comparable to those of meat patties formulated with full pork backfat. These results indicate that 25% and 50% replacement levels have considerable potential for practical fat substitution in emulsified meat patties products.

## 4 Conclusion

In the present study, HIPPE and HIPPE gels was succesfully constructed with modified protein-polysaccharide composite particles and flaxseed oil. Particle characterization revealed that proteins rich in hydrophobic amino acids interacted with hydrophilic sodium alginate via electrostatic interactions, resulting in the formation of amphiphilic composite particles. Moreover, ultrasonic treatment was indispensable for particle formation, as it markedly reduced particle size and optimized the zeta potential, thereby ensuring that the resulting particles met the requirements for effective Pickering emulsion stabilization. Compared with unmodified particles, HIPPE with succinylated composite particles exhibited faster interfacial adsorption, lower interfacial tension, and stronger resistance to droplet coalescence. Obviously, succinylation markedly improves their interfacial and stabilizing performance. In addition, the emulsions exhibited distinct pH and thermal responsiveness, demonstrating their sensitivity to environmental changes and confirming the tunable nature of their structural stability. Under the optimized conditions, the HIPPE gels was prepared. Notably, following Ca^2+^-induced gelation, the HIPPE gels developed a microstructure that closely resembled that of animal fat tissue. Texture, color, and sensory analyses of meat patties prepared with different levels of fat susititute also showed that meat patties with 25% and 50% substitution exhibited no significant differences from those containing 100% pork back fat (*P* > 0.05).

This technology offers several notable advantages, including improved interfacial stability, high internal oil-phase loading, structural tunability, and promising potential for application as a sustainable fat analogue. However, some limitations should be acknowledged. The capacity of the HIPPE gels to deliver bioactive compounds has not yet been investigated. In addition, although the gels system showed promising textural properties, it still cannot fully replicate the sensory and textural characteristics of animal fat. Furthermore, more marine-based macromolecular materials need to be explored and systematically elucidated to optimize the design of such fat analogues. Future studies should place greater emphasis on incrasing the sensory, texture and optimizing the choose of marin-based protein of HIPPE gels. Overcoming these limitations will be crucial for advancing their commercial translation and expanding their applications in Pickering systems for fat substitution, lipophilic bioactive delivery, as well as in food, pharmaceutical, and other soft-material-related fields.

## CRediT authorship contribution statement

**Xinxin Si:** Writing – original draft, Investigation, Formal analysis, Data curation. **Zhuozhen Li:** Investigation, Formal analysis, Data curation. **Yixi Chen:** Validation, Investigation. **Bingheng Li:** Formal analysis. **Li Zhang:** Validation. **Jiefu Hai:** Conceptualization. **Yichen Zhang:** Methodology. **Boyao Su:** Software. **Xiao Feng:** Writing – review & editing. **Tian Zhong:** Visualization, Validation, Supervision. **Ying Xiao:** Supervision, Formal analysis, Conceptualization. **Xi Yu:** Writing – review & editing, Supervision, Resources, Project administration, Funding acquisition, Conceptualization.

## Declaration of competing interest

The authors declare that they have no known competing financial interests or personal relationships that could have appeared to influence the work reported in this paper.

## Data Availability

Data will be made available on request.

## References

[1] Zembyla M., Murray B.S., Sarkar A. (2020). Water-in-oil emulsions stabilized by surfactants, biopolymers and/or particles: a review. Trends Food Sci Tech..

[2] Guo Y., Luo Y., Liu X., Lai H., Liu Y., Cui X., Dong M., Luo X., Wu C., Yu X. (2026). The association between food group-based inflammatory score and cardiometabolic disorders in Ganzhou City of China. Sci. Rep..

[3] Xie P., Mao Y., Zou S., Ke W., Lee Y.-Y., Tan C.-P., Wang Y., Zhang Z. (2025). Potential of diacylglycerol to replace nature fats in conventional plastic fats: preparation, purification, functionalities and applications. Trends Food Sci Tech..

[4] Xu Q., Wang H., Ren Y., Sun M., Zhang T., Li H., Liu X. (2024). Functionality and application of emulsion gels in fat replacement strategies for dairy products. Trends Food Sci Tech..

[5] Guo Y., Zhang Y., Han L., Wang Y., Zhong T., Xiao Y., Lam L.T., Wu C., Yu X. (2025). Study on fatigue, sleep quality and PTSD symptoms and influencing factors among prevention and control workers during the COVID-19 outbreak in China. Sci. Rep..

[6] S.U. Pickering, CXCVI.—Emulsions, J. Chem. Soc., Perkin Trans. 91 (1907) 2001-2021, doi:10.1039/CT9079102001.

[7] Huang Y., Ma T., Li C., McClements D.J. (2026). Plant-based emulsion gels assembled from potato protein, β-glucan and olive oil: structure, functionality, and application as low-fat butter replacers. Food Hydrocoll..

[8] Nimaming N., Sadeghpour A., Murray B.S., Sarkar A. (2023). Hybrid particles for stabilization of food-grade Pickering emulsions: fabrication principles and interfacial properties. Trends Food Sci. Technol..

[9] Guo Z., Yan Z., Chen Y., Ieong W.I., Zhong T., Xiao Y., Yu X. (2025). Protective role of natural saccharides in frozen animal food formulations: enhancing cryopreservation during cold treatment. Int. J. Biol. Macromol..

[10] Huang J., Quan W., Lou A., Luo J., Fan X., Shen Q., Zhou L. (2026). Applying polysaccharides to enhance the quality of protein-based emulsion gels: a comprehensive review. J. Future Foods..

[11] Yun Z., Dong Y., Liu Z., Yu X., Xiao Y., Fang Q., Zhong T. (2026). The “Bridge” between hydrophobic active substances and hydrophilic biopolymers: the role of cyclodextrins in enhancing the properties of food packaging films. Trends Food Sci. Technol..

[12] He Y., Li Z., Feng X., Liang D., Liu L., He G., Zhong T., Xiao Y., Yu X. (2026). Carbon quantum dots in food analysis: challenges in real matrices, recognition mechanisms, and optimization strategies. Food Biosci..

[13] Li L., Li Z., Balle T., Liu G., Guo Z. (2023). Biosynthesis of pectic oligosaccharide-based amphiphiles as novel stabilizers of nanoemulsions by coupling enzymatic depolymerization with alkyl/alkenyl succinylation. Food Hydrocoll..

[14] Si X., Si Y., Lu Z., Zhong T., Xiao Y., Wang Z., Yu X. (2025). Mechanisms of fatigue and molecular diagnostics: the application of bioactive compounds in fatigue relief research. Food Biosci..

[15] A. Simovic, S. Combet, T. Cirkovic Velickovic, M. Nikolic, S. Minic, Probing the stability of the food colourant R-phycoerythrin from dried Nori flakes, Food Chem. 374 (2022) 131780, doi:10.1016/j.foodchem.2021.131780. 10.1016/j.foodchem.2021.131780 34894468

[16] L. Veličković, A. Simović, N. Gligorijević, A. Thureau, M. Obradović, T. Vasović, G. Sotiroudis, M. Zoumpanioti, A. Brûlet, T. Ćirković Veličković, S. Combet, M. Nikolić, S. Minić, Exploring and strengthening the potential of R-phycocyanin from Nori flakes as a food colourant, Food Chem. 426 (2023) 136669, doi:10.1016/j.foodchem.2023.136669. 10.1016/j.foodchem.2023.136669 37352716

[17] Liu Y., Zhang H., Zhang Z., Dong Y., Jiang T., Zhang Y., Peng Y., Yu X., Xiao Y., Zhong T. (2025). Food contact materials based on N-halamines or photosensitizers: an emerging strategy for developing “rechargeable” antibacterial properties. Trends Food Sci. Technol..

[18] Zhang J., Tang J., Shi S., Huang H., Li Y., Liu W., Shi J., Tong C., Pang J., Wu C. (2025). Research progress on marine polysaccharide-based Pickering emulsions and their potential applications in the food industry. Food Res. Int..

[19] Meng W., Sun H., Mu T., Garcia-Vaquero M. (2024). Spray-drying and rehydration on β-carotene encapsulated Pickering emulsion with chitosan and seaweed polyphenol. Int. J. Biol. Macromol..

[20] S. Ghelichi, B. Shokrollahi Yancheshmeh, M.K.T. Hong Lin, C. Jacobsen, Fabrication and characterization of high internal phase Pickering emulsions (HIPPEs) of ω-3 algal oil stabilized with seaweed-derived particles, Food Chem. 498 (2026) 147073, doi:10.1016/j.foodchem.2025.147073. 10.1016/j.foodchem.2025.147073 41265088

[21] Hu X., Jiang Q., Du L., Meng Z. (2023). Edible polysaccharide-based oleogels and novel emulsion gels as fat analogues: a review. Carbohydr. Polym..

[22] Xu F.-Y., Cai J.-L., Chen B.-R., Zeng X.-A., Wang L.-H., Wang R. (2025). A bilayer emulsion gel stabilized by succinylated whey protein isolate and chitosan for delivering curcumin: characterization, stability, and digestive properties. Int. J. Biol. Macromol..

[23] Si X., Si Y., Zhang S., Liu Y., Liu Y., Wang H., Wang Z. (2023). Effect of grain/bran crude extract from Fagopyrum tataricum on hypoglycemic activity of type 2 diabetes mice and study on molecular mechanism of treatment. Cogent Food Agric..

[24] Li F., Huang L., Yuan C., Dai M., Yang X., Lang Y. (2026). High internal phase Pickering emulsion stabilized by succinylation of myofibrillar protein for β-carotene delivery. Food Chem..

[25] K.e. Xiao, L. Gao, Q. Lu, Q. Wang, Z. Zhao, M. Sai, X. Meng, L. Guan, J. Yang, L. Du, Development and characterization of high internal phase emulsions for lycopene encapsulation with safflower seed meal globulin modified via ultrasound, heating and pH-shifting, Ultrason. Sonochem. 128 (2026) 107805, doi:10.1016/j.ultsonch.2026.107805. 10.1016/j.ultsonch.2026.107805 PMC12992074 41795413

[26] Huang Y., Zheng B., Sun B., Zhu Y., Liu L., Liu J., Zhang Y., Li Y., Zhu X. (2025). Mechanism of emulsion stabilized by an ultrasonically prepared protein–polyphenol-polysaccharide complex: structure, functional properties and interfacial behavior. Ultrason. Sonochem..

[27] W. Zhu, Y. Dong, T. Wu, H. Jing, Z. Li, X. Yu, Y. Xiao, T. Zhong, Beta-cyclodextrin inclusion complexes of citral and linalool inhibit *Escherichia coli* on cooked chicken: Focus on their synergistic antibacterial effects, Food Chem.: X. 32 (2025) 103248, doi:10.1016/j.fochx.2025.103248. 10.1016/j.fochx.2025.103248 PMC12648581 41312203

[28] Lian Z., Yang S., Cheng L., Liao P., Dai S., Tong X., Tian T., Wang H., Jiang L. (2023). Emulsifying properties and oil–water interface properties of succinylated soy protein isolate: affected by conformational flexibility of the interfacial protein. Food Hydrocoll..

[29] Ma L., Li J., Liu Y., Zheng J. (2025). Ultrasonic engineering of bovine serum albumin nanoparticles for high internal phase Pickering emulsions: interfacial behavior, microstructural evolution and stabilization enhancement. Ultrason. Sonochem..

[30] Zhou X., Zhang B., Huang W. (2024). Carboxymethyl chitosan and dialdehyde cellulose nanocrystal based injectable self-healing emulsion gel. Carbohydr. Polym..

[31] Wu B., Yang C., Xin Q., Kong L., Eggersdorfer M., Ruan J., Zhao P., Shan J., Liu K., Chen D., Weitz D.A., Gao X. (2021). Attractive Pickering Emulsion Gels. Adv. Mater..

[32] Zhang J., Zheng Y., Guo B., Sun D., Xiao Y., Yang Z., Liu R., Chen J., Wu B., Zhao P., Ruan J., Weitz D.A., Chen D. (2024). Jammed Pickering Emulsion Gels. Adv. Sci..

[33] Zhou C., Feng Y., Chen R., Li J., Zhang J., Jia J., Yan J., Peng X., Dong Z., Wu Z. (2026). Ultrasonic microreactor-mediated fabrication of stable W1/O/W2 double emulsions for efficient vitamin C encapsulation. Ultrason. Sonochem..

[34] Huang Y., Wang Z., Lu Y., Zhu B., Liu J., Qi B., Sun B. (2026). Stabilization and delivery performance of soybean oil body emulsions: the role of chitosan, guar gum, and ι-carrageenan in polysaccharide-based systems. Ultrason. Sonochem..

[35] Bai Y., Shu X., Ai Y., Wang H., Hou W., Han Y. (2026). Effects of whole lotus root powder particle size on gel properties and microstructure of duck emulsified sausage: underlying mechanisms of gelling behavior. Food Chem..

[36] Zhuang X., Han M., Kang Z.-L., Wang K., Bai Y., Xu X.-L., Zhou G.-H. (2016). Effects of the sugarcane dietary fiber and pre-emulsified sesame oil on low-fat meat batter physicochemical property, texture, and microstructure. Meat Sci..

[37] Zhang J., Li D., Zhang Y., Tang J., Shi S., Zeng X., Chen H., Pang J., Wu C. (2025). The effects of soy protein isolate-based composite gels as pork back fat substitutes in low-fat emulsified sausage. Food Res. Int..

[38] Nie C., Zhong H., Shi H., Yao X., Wang W., Tomasevic I., Zheng J., Sun W. (2026). Sodium substitutes synergizing with dietary fiber: effect on the textural, sensory, digestive, and storage properties of emulsified sausages. Food Chem..

[39] N. Dnyaneshwar Patil, A. Bains, S. Kaur, R. Yadav, N. Ali, S. Patil, G. Goksen, P. Chawla, Influence of dual succinylation and ultrasonication modification on the amino acid content, structural and functional properties of Chickpea (*Cicer arietinum* L.) protein concentrate, Food Chem. 445 (2024) 138671, doi:10.1016/j.foodchem.2024.138671. 10.1016/j.foodchem.2024.138671 38367556

[40] Quaisie J., Ma H., Yiting G., Tuly J.A., Igbokwe C.J., Zhang X., Ekumah J.-N., Akpabli-Tsigbe N.D.K., Nianzhen S. (2021). Impact of sonication on slurry shear -thinning of protein from sea cucumber (Apostichopus japonicus): proteolytic reaction kinetics, thermodynamics, and conformational modification. Innovative Food Sci. Emerg. Technol..

[41] Lee H.G., Lee J., Jo Y.-J., Choi M.-J. (2025). Ultrasonication time dependent structuring of heat-treated legume proteins: interfacial adsorption and stabilization of faba bean and pea protein isolates in high internal phase Pickering emulsions. Ultrason. Sonochem..

[42] Gao K., Rao J., Chen B. (2022). Unraveling the mechanism by which high intensity ultrasound improves the solubility of commercial pea protein isolates. Food Hydrocoll..

[43] Liu G., Hu M., Du X., Qi B., Lu K., Zhou S., Xie F., Li Y. (2022). Study on the interaction between succinylated soy protein isolate and chitosan and its utilization in the development of oil-in-water bilayer emulsions. Food Hydrocoll..

[44] Nie H.-N., Dong H., Chen Y.-L., Hao M.-M., Chen J.-N., Tang Z.-C., Liu Q.-Z., Li J.-K., Xu X.-B., Xue Y.-L. (2023). Effects of spray drying and freeze drying on the structure and emulsifying properties of yam soluble protein: a study by experiment and molecular dynamics simulation. Food Chem..

[45] Wang Y., Ge S., Li X., Zhang K., Li M., Sun Q., Xie F., Ji N., Qin Y. (2026). One-step Fabrication of Dual-Network Corn Starch–Sodium Alginate Emulsion Gels to Improve Curcumin Stability and Bioaccessibility. J. Future Foods..

[46] Han G., Han A., Chang Y.H. (2026). Effect of carboxymethyl cellulose on structural, physicochemical, and textural properties of faba bean protein/carboxymethyl cellulose emulsion gel as a plant-based ricotta cheese analog. Food Hydrocoll..

[47] Jing H., Dong Y., Wu T., Zhu W., Li Z., Jiang T., Liu Y., Sui S., Yu X., Xiao Y., Zhong T. (2025). An innovative sodium carboxymethyl cellulose-based chlorine dioxide-releasing hydrogel incorporating α-cyclodextrin inclusion complexes and its potential for extending fruit shelf-life. Int. J. Biol. Macromol..

[48] Yin C., Chen X., Zhang H., Xue Y., Dong H., Mao X. (2024). Pickering emulsion biocatalysis: bridging interfacial design with enzymatic reactions. Biotechnol. Adv..

[49] Yu J., Li J., Xu D., Gao Y., Mao L. (2025). Insights into the stabilization and rheological properties of water-in-oil high internal phase emulsions based on κ-carrageenan hydrogels. Food Res. Int..

[50] Choi M., Choi H.W., Kim H.E., Hahn J., Choi Y.J. (2023). Mimicking animal adipose tissue using a hybrid network-based solid-emulsion gel with soy protein isolate, agar, and alginate. Food Hydrocoll..

[51] Zhao J., Chang B., Hu Y., Wang X., Cao Z., Zhang Y., Xu Z., Sui X. (2025). Adsorption mechanism of soy protein amyloid fibrils with different morphological structures at the interface of oil-in-water emulsion. Food Hydrocoll..

[52] Yao W., Liu Y., Kong B., Sun F., Zhang H., Liu Q., Cao C. (2026). Understanding the enhancement effect of emulsion stability and gelling properties of myofibrillar protein emulsions stabilized with κ-carrageenan: perspective on rheological behaviors and intermolecular forces. Food Hydrocoll..

[53] Guo J., Cui L., Huang Y., Meng Z. (2022). Spirulina platensis protein isolate nanoparticle stabilized O/W Pickering emulsions: interfacial adsorption and bulk aggregation. Food Res. Int..

[54] Xue Y., Song J., Chen S., Fu C., Li Z., Weng W., Shi L., Ren Z. (2025). Improving surimi gel quality by corn oligopeptide-chitosan stabilized high-internal phase Pickering emulsions. Food Hydrocoll..

[55] Li Q., Weng W., Shi L., Huang W., Wang Y., Chen Y., Ren Z. (2026). Novel Pickering emulsions stabilized by abalone oligopeptide and xanthan gum complexes: effects of concentrations and oil-water ratios. Food Hydrocoll..

[56] Wang J., Wang Z., Chen S., Liu S., Li C., Sun Y. (2026). [BMIM]Cl concentration-regulated wet ball milling of coconut pomace cellulose: structural modification-driven enhancement of Pickering emulsion stability. J. Future Foods..

[57] Liu Z., Shen R., Yang X., Lin D. (2021). Characterization of a novel konjac glucomannan film incorporated with Pickering emulsions: effect of the emulsion particle sizes. Int. J. Biol. Macromol..

[58] Guo J., Liao H., Zhang J., Qiu Y., Xue C., Zhu J. (2025). Hemp seed oil-based emulsion gels stabilized by soybean protein isolate, inulin, and glyceryl monostearate: phase inversion and baking application. Int. J. Biol. Macromol..

[59] Niroula A., Poortinga A.T., Nazir A. (2025). Pickering stabilization of double emulsions: basic concepts, rationale, preparation, potential applications, challenges, and future perspectives. Adv. Colloid Interface Sci..

[60] Zhu L., Liao H., Zhuang K., Sang S., Chen L., Chang X., Zhang Q., Lv Q., Liu X., Liu X., Ding W. (2025). Mechanism of ultrasonic-alkali-thermal modification for enhancing the emulsifying properties of rice protein and its stability in high-internal-phase emulsions. Ultrason. Sonochem..

[61] Liu Z., Li B., Du Y., Jia W., Belwal T., Wu H., Jin X., Fu X. (2025). Lycopene W/O/W emulsions stabilized by *Cyperus esculentus* starch: effect of oil phase content on stability and in vitro digestive behavior. LWT-Food Sci Technol..

[62] Li X., Fan L., Li J. (2023). Utilization of polysaccharide-based high internal phase emulsion for nutraceutical encapsulation and 3D printing: reinforcement of curcumin stability and bioaccessibility. LWT-Food Sci Technol..

[63] Lao Y., Wang Y., Selomulya C. (2024). Exploring the oil-water interfacial behaviour of pea protein-guar gum/gellan gum mixtures relating to stability and in vitro protein digestion of model pea milk system. Int. J. Biol. Macromol..

[64] Dong Z., Zhang F., Wang P., Xu X. (2026). Ultrasound-assisted tannic acid crosslinking of myofibrillar protein-stabilized high internal phase emulsions: enhanced stability, mechanical properties, and 3D printability. Ultrason. Sonochem..

[65] Zhao T., Huang K., Luo Y., Li Y., Cheng N., Mei X. (2023). Preparation and characterization of high internal phase Pickering emulsions stabilized by hordein-chitosan composite nanoparticles. Colloids Surf A Physicochem Eng Asp.

[66] Chen Z., Wang A., Qin Y., Chen X., Feng X., He G., Zhu X., Xiao Y., Yu X., Zhong T., Zhang K. (2024). Preparation of a thermosensitive and antibacterial in situ gel using poloxamer-quaternized chitosan for sustained ocular delivery of Levofloxacin hydrochloride. Int. J. Biol. Macromol..

[67] Sun F., Cheng T., Ren S., Yang B., Liu J., Huang Z., Guo Z., Wang Z. (2024). Soy protein isolate/carboxymethyl cellulose sodium complexes system stabilized high internal phase Pickering emulsions: stabilization mechanism based on noncovalent interaction. Int. J. Biol. Macromol..

[68] Zhang T., Wu Z., Zhu H., Wang Z., Sun S., Hu S. (2023). pH/temperature-responsive salt-tolerant Pickering emulsion formed by PNIPAM-modified chitosan particles. Colloids Surf A Physicochem Eng Asp.

[69] Shen Y., Li Y. (2021). Acylation modification and/or guar gum conjugation enhanced functional properties of pea protein isolate. Food Hydrocoll..

[70] Li D., Wang Y., Liang S., Bai B., Zhang C., Xu N., Shi W., Ding W., Zhang Y. (2024). Synthesizing dendritic mesoporous silica nanoparticles to stabilize Pickering emulsions at high salinity and temperature reservoirs. Colloids Surf A Physicochem Eng Asp.

[71] Du L., Ru Y., Weng H., Zhang Y., Xiao A., Chen J., Xiao Q. (2025). Agar-lauric acid complex-stabilized Pickering emulsions: fabrication, stabilization, and probiotics encapsulation. Food Hydrocoll..

[72] Wang L., Yu X., Cheng C., Shao J., Xu J., Xiang X., Chen L., Deng Q. (2026). Ratio-dependent engineering of sunflower protein–phospholipid interfaces in flaxseed oil-in-water emulsions: toward enhanced α-linolenic acid bioaccessibility. J. Future Foods..

[73] Lee S., Jo K., Kim S., Woo M., Choi Y.-S., Jung S. (2025). Constructing stable emulsion gel from gelatin and sodium alginate as pork fat substitute: emphasis on lipid digestion in vitro. Food Hydrocoll..

[74] Choi Y.-S., Choi J.-H., Han D.-J., Kim H.-Y., Lee M.-A., Kim H.-W., Jeong J.-Y., Kim C.-J. (2011). Effects of rice bran fiber on heat-induced gel prepared with pork salt-soluble meat proteins in model system. Meat Sci..

[75] Zhang Z., Zhang Y., Ah Y., Liu S., Xu J., Liu Q., Kong B., Sun F. (2025). Physical properties and structural modifications of myofibrillar gel prepared with Lycium barbarum polysaccharide under low-salt conditions and its application in Frankfurter sausage production. Food Chem..

[76] Zhang A., Yang B., Xu S., Liu K., Dong H., Pan J., Liao W., Yang X., He Q. (2025). Emulsion gels based on soy protein–pectin complexes as fat replacers: enhancing pork sausage quality through structural and molecular insights. Int. J. Biol. Macromol..

[77] Ma C.-Y., Wang C., Wang X.-M., Liu J., Tu Z.-C., Shao Y.-H. (2026). Dynamic ultra-high pressure modified soy protein isolate: mechanism on the stabilization of high internal phase emulsions and its application as pork sausages. Food Chem..

[78] Nacak B., Öztürk-Kerimoğlu B., Yıldız D., Çağındı Ö., Serdaroğlu M. (2021). Peanut and linseed oil emulsion gels as potential fat replacer in emulsified sausages. Meat Sci..

[79] Lee H.J., Han J.H., Keum D.H., Kothuri V., Shin D.-M., Han S.G. (2025). Quercetin-loaded candelilla wax/sunflower oil oleogels: structural, sensory, and storage properties, and application as fat replacer in emulsion-type sausage. Food Chem..

