# Supplementary material for: Succinylated nori protein–alginate complex particles prepared using temperature-controlled ultrasonication for the formation of high internal phase Pickering emulsion gels as plant-based fat analogues
Source: Ultrason Sonochem. 2026 Aug 26;133:108029. doi: 10.1016/j.ultsonch.2026.108029 (PMC13559836; doi:10.1016/j.ultsonch.2026.108029)
Supplement: Supplementary file 1 [file mmc1.docx]

# 2 Materials and methods

## 2. 1 Extraction of Nori Proteins

Nori was soaked in 0.1 M NaOH and subjected to ultrasound-assisted extraction for 6 h at 55-60 °C. After filtration, the supernatant was adjusted to pH 4.5 with 1 mol/L HCl and precipitated overnight at 4 °C. The precipitate was collected by centrifugation (8000× g, 15 min) and freeze-dried. Protein concentration was determined by the Lowry method using BSA as a standard [1].

## 2.2 Analysis of amino acid composition in nori protein

Automated pre-column derivatization amino acid analysis was conducted on a Shimadzu LC-20A HPLC system using an AJS-01 C18 column (3 μm, 4.6 × 150 mm) at 50 °C. Mobile phase A was phosphate-borate buffer (pH 8.2); mobile phase B was methanol:acetonitrile:water (4.5:4.5:1, v/v/v). For derivatization, 50 μL borate buffer, 20 μL sample, 10 μL OPA (0.5 min reaction), and 10 μL FMOC (1 min reaction) were sequentially mixed, and 9 μL was injected. Quantification was based on calibration curves (0.02–0.5 μmol/L) using 16 amino acid standards (WAT 700005089).

## 2.3 Determination of the Degree of Succinylation（DS）

Nori protein was succinylated using varying amounts of succinic anhydride (1–20%, w/w protein) following the method. The degree of substitution (DS) was determined by the ninhydrin assay [2]. Briefly, 1% (w/w) protein was mixed with equal volume of 0.1% ninhydrin, boiled for 15 min, cooled, and diluted six-fold with water. Absorbance at 570 nm was measured (UV-2600, Shimadzu), and DS was calculated using Equation (1).

DS（％）=$\frac{C0-C1}{C0}$×100	(1)

where C₀ and C₁ represent the absorbance values of the control and succinylated samples, respectively.

## 2.4 Particle Size and Zeta Potential of NP, SNP and SA

The zeta potential and particle size distribution of the particles were determined using a Zetasizer Laboratory instrument (model Fe-U0801, Malvern Panalytical, UK).

## 2.5 Height-based index of stratification (HIS) and Creaming index (CI)

To evaluate the stability of the Pickering emulsions, samples were stored at 4 °C and photographed at predetermined time intervals. HIS was calculated using Equation (1). Centrifugation stability was assessed by preparing the emulsions in centrifuge tubes and centrifuging at 1500 × g for 10 min. Following centrifugation, images were captured, and CI was determined according to Equation (2) to evaluate stability [3, 4].

HIS(%)=$\frac{H_{S}}{H_{e}}$	(2)

$CI(\%)=\frac{H_{s}}{H_{t}}*100$	(3)

where Hₛ is the height of the lower aqueous phase, Hₑ is the total height of the emulsion and Hₜ is the total height of the emulsion before centrifugation.

## 2.6 Emulsion stability index (ESI) and emulsion activity index (EAI)

The emulsifying properties were determined according to a modified method described by Yao et al. Briefly, 100mg of emulsion was diluted 10-fold with 0.1% (w/w) sodium dodecyl sulfate (SDS) solution. Then, 60 μL of the diluted emulsion was mixed uniformly and further diluted to 3mL with the same SDS solution. Absorbance was measured at 500 nm using a spectrophotometer (UV‑6100, MAPADA, Shanghai, China), with 0.1% SDS solution serving as the blank. The emulsion activity index (EAI) and emulsion stability index (ESI) were calculated using the following equations (3) and (4) [5].

EAI(m^2^/g)=$\frac{2\times2.303\times A_{0}\times N}{C\timesФ\times10000}$	(4)

ESI(%)=$\frac{A_{10}}{A_{0}}\times100\%$	(5)

where，$A_{0}$is the absorbance immediately after emulsion formation；$A_{10}$ is the absorbance after 10 min，N is the dilution factor，C is The concentration of particles (in the continuous phase) (g/mL)，$Ф$ is the oil volume fraction.

## 2.7 Texture profile analysis(TPA) of high internal phase Pickering emulsions (HIPPE)

Texture profile analysis (TPA), stress relaxation, and creep tests were performed using a Brookfield texture analyzer (TA-CT3). For TPA, samples (15 cm height in 100 mL beakers) were compressed twice with a 30 mm cylindrical probe (TA3/100) at 40% deformation and 20 mm/min (trigger force: 0.15 N). All tests were carried out in triplicate, and the mean values with corresponding standard deviations were calculated. Stress relaxation was measured at 50% deformation (20 mm/min, trigger force 0.15 N). Creep tests were conducted under a constant stress of 1 N for 180 s (initial force 0.05 N, pre-test speed 20 mm/min). In both tests, data recording commenced immediately upon probe contact with the sample surface.

# References

[1] N.B. Rizvi, S. Aleem, M.R. Khan, S. Ashraf, R. Busquets, Quantitative Estimation of Protein in Sprouts of Vigna radiate (Mung Beans), Lens culinaris (Lentils), and Cicer arietinum (Chickpeas) by Kjeldahl and Lowry Methods, Molecules. 27 (2022) 814, https://doi.org/10.3390/molecules27030814.

[2] Z. Lian, S. Yang, L. Cheng, P. Liao, S. Dai, X. Tong, T. Tian, H. Wang, L. Jiang, Emulsifying properties and oil–water interface properties of succinylated soy protein isolate: Affected by conformational flexibility of the interfacial protein, Food Hydrocoll. 136 (2023) 108224, https://doi.org/10.1016/j.foodhyd.2022.108224.

[3] J. Zhao, X. Feng, J. Han, D. Liu, X. Yang, D. Lin, The construction of pH responsive O/W1/W2 double emulsions based on carboxymethyl cellulose and chitosan: Structure, rheological behaviors and stability, Int. J. Biol. Macromol. 320 (2025) 145571, https://doi.org/10.1016/j.ijbiomac.2025.145571.

[4] I. Nasim, N. Khalid, Enhanced extraction of beetroot peel polyphenols using response surface methodology and their stabilization in W/O/W emulsions using whey protein isolates, Ultrason. Sonochem. 123 (2025) 107638, https://doi.org/10.1016/j.ultsonch.2025.107638.

[5] B. Cui, Y. Zhang, C. Zhu, K. Li, Y. Wang, Y. Wang, W. Ma, Y. Bai, Ultrasound-mediated fabrication of chickpea protein nanoparticles for stabilizing Pickering emulsions, Ultrason. Sonochem. 121 (2025) 107574, https://doi.org/10.1016/j.ultsonch.2025.107574

**Tables:**

Table S1. The texture profile analysis of the HIPPE.

| Result Info. | Units | NSE | SSE |
| --- | --- | --- | --- |
| Hardness | N | 1.32±0.16 | 2.44±0.22 |
| Adhesive Force | N | -0.88±0.16 | -1.82±0.21 |
| Adhesiveness | N.mm | 2.90±0.54 | 6.51±0.73 |
| Cohesiveness | Ratio | 0.88±0.04 | 0.78±0.15 |
| Springiness | mm | 3.77±0.22 | 5.68±0.52 |
| Gumminess | N | 1.17±0.17 | 1.88±0.22 |
| Chewiness | mj | 4.42±0.89 | 10.76±2.04 |

**Tabl**e S2 Formulation of meat patties with HIPPE gels instead of pork fat.

| **Ingredients** | **T1** | **T2** | **T3** | **T4** | **T5** |
| --- | --- | --- | --- | --- | --- |
| Lean pork meat | 80 | 80 | 80 | 80 | 80 |
| Pork back fat | 20 | 15 | 10 | 5 | 0 |
| Fat substitute | 0 | 5 | 10 | 15 | 20 |
| Corn starch | 4 | 4 | 4 | 4 | 4 |
| Salt | 2 | 2 | 2 | 2 | 2 |
| Sugar | 0.8 | 0.8 | 0.8 | 0.8 | 0.8 |
| Sodium Tripolyphosphate | 0.2 | 0.2 | 0.2 | 0.2 | 0.2 |
| Sodium D-isoascorbate | 0.5 | 0.5 | 0.5 | 0.5 | 0.5 |
| Red yeast rice Pigment | 0.0025 | 0.0025 | 0.0025 | 0.0025 | 0.0025 |
| Ice water | 20 | 20 | 20 | 20 | 20 |
| pepper | 0.24 | 0.24 | 0.24 | 0.24 | 0.24 |
| Monosodium glutamate | 0.48 | 0.48 | 0.48 | 0.48 | 0.48 |
| Light soy sauce | 1.6 | 1.6 | 1.6 | 1.6 | 1.6 |
| Onion | 20 | 20 | 20 | 20 | 20 |

Fig. S1. Characterization of Nori protein and its succinylated derivatives. (A) Standard curve for protein content quantification. (B) Determination of succinylation degree in Nori protein. (C) The amino acid content in NP and SNP. (D) The Zeta potentials of NP, SNP and SA at different PH values. (NP: native/succinylated nori protein. SNP: Succinylated nori protein. SA: Sodium alginate ).

Fig. S2. Characterization of emulsions prepared with different particle contents. (A) EAI and ESI of Pickering emulsions formed with different particle contents (in the continuous phase). (B) Centrifugal stability of PEs formed with different particle contents (in the continuous phase). (C) HSI values of NSE with different particle contents (in the continuous phase). (D) HSI values of SSE with different particle contents (in the continuous phase). (E) Visual appearance of NSE with different particle contents after 10 days of storage. (F) Visual appearance of SSE with different particle contents after 10 days of storage. (**NSE**, emulsion formed by natural protein and sodium alginate complex particles. **SSE**, emulsion formed by succinylated protein and sodium alginate complex particles).

Fig. S3 Characterization of emulsions prepared with different protein-to-polysaccharide ratio. (A) Emulsion activity index (EAI) and emulsion stability index (ESI) of emulsions formulated with different protein-to-polysaccharide mass ratios. **(B)** Centrifugal stability（CI） of emulsions prepared with varying protein-to-polysaccharide ratios. **(C)** Height-based index of stratification (HIS) values of NSE of different protein-to-polysaccharide ratio. **(D)** HIS values of SSE of different protein-to-polysaccharide ratio. **(E)** Visual appearance of NSE with different protein-to-polysaccharide ratios after 10 days of storage. **(F)** Visual appearance of SSE with different protein-to-polysaccharide ratios after 10 days of storage. (NSE, emulsion formed by natural protein and sodium alginate complex particles. SSE, emulsion formed by succinylated protein and sodium alginate complex particles).

**Fig. S4.** Characterization of emulsions prepared with different oil phase ratios. **(A)** Emulsion activity index (EAI) and emulsion stability index (ESI) of emulsions formulated with different oil phase ratios. **(B)** Centrifugal stability oil phase ratios of emulsions formulated with different oil phase ratios. **(C)** Height-based index of stratification (HIS) values of NSE of different oil phase ratios. **(D)** Height-based index of stratification (HIS) values of SSE of different oil phase ratios. **(E)** Visual appearance of NSE with different oil phase ratios after 10 days of storage. **(F)** Visual appearance of NSE with different oil phase ratios after 10 days of storage. (NSE, emulsion formed by natural protein and sodium alginate complex particles. SSE, emulsion formed by succinylated protein and sodium alginate complex particles).

Fig. S5 the centrifugal stability of pickering emulsion and the machanism of stability in different conditions.

Fig. S6  Microscopic observation and Visual appearance of HIPPE in the different temperatures and pH (A) Microstrutures of the temperature responsiveness of HIPPE. (B) Microstrutures of pH responsiveness of HIPPE.(C) Visual appearance of HIPPE in the different temperatures and pH.
